# Targeting pyruvate dehydrogenase kinase 1 overcomes *EGFR* C797S mutation-driven osimertinib resistance in non-small cell lung cancer

**DOI:** 10.1038/s12276-024-01221-2

**Published:** 2024-05-01

**Authors:** Wonyoung Park, Shibo Wei, Chu-Long Xie, Jung Ho Han, Bo-Sung Kim, Bosung Kim, Jung-Sook Jin, Eun-Sun Yang, Min Kyoung Cho, Dongryeol Ryu, Hao-Xian Yang, Sung-Jin Bae, Ki-Tae Ha

**Affiliations:** 1 Department of Korean Medical Science, School of Korean Medicine, Pusan National University, Yangsan, Gyeongsangnam-do 50612 Republic of Korea; 2 Korean Medical Research Center for Healthy Aging, Pusan National University, Yangsan, Gyeongsangnam-do 50612 Republic of Korea; 3 Department of Precision Medicine, School of Medicine, Sungkyunkwan University School of Medicine, Suwon, Gyeonggi-do 16419 Republic of Korea; 4 Department of Thoracic Surgery, Sun Yat-sen University Cancer Center, Guangzhou, People’s Republic of China; 5 State Key Laboratory of Oncology in South China, Collaborative Innovation Center for Cancer Medicine, Sun Yat-sen University Cancer Center, Guangzhou, People’s Republic of China; 6 Korean Medicine Application Center, Korea Institute of Oriental Medicine, Daegu, 41062 Republic of Korea; 7 Department of Molecular Biology and Immunology, Kosin University College of Medicine, Busan, 49267 Republic of Korea; 8 Department of Biomedical Science and Engineering, Gwangju Institute of Science and Technology, Gwangju, 61005 Republic of Korea

**Keywords:** Non-small-cell lung cancer, Non-small-cell lung cancer

## Abstract

Osimertinib, a selective third-generation epidermal growth factor receptor (EGFR) tyrosine kinase inhibitor (TKI), effectively targets the *EGFR* T790M mutant in non-small cell lung cancer (NSCLC). However, the newly identified *EGFR* C797S mutation confers resistance to osimertinib. In this study, we explored the role of pyruvate dehydrogenase kinase 1 (PDK1) in osimertinib resistance. Patients exhibiting osimertinib resistance initially displayed elevated PDK1 expression. Osimertinib-resistant cell lines with the *EGFR* C797S mutation were established using A549, NCI-H292, PC-9, and NCI-H1975 NSCLC cells for both in vitro and in vivo investigations. These *EGFR* C797S mutant cells exhibited heightened phosphorylation of EGFR, leading to the activation of downstream oncogenic pathways. The *EGFR* C797S mutation appeared to increase PDK1-driven glycolysis through the EGFR/AKT/HIF-1α axis. Combining osimertinib with the PDK1 inhibitor leelamine helped successfully overcome osimertinib resistance in allograft models. CRISPR-mediated PDK1 knockout effectively inhibited tumor formation in xenograft models. Our study established a clear link between the *EGFR* C797S mutation and elevated PDK1 expression, opening new avenues for the discovery of targeted therapies and improving our understanding of the roles of *EGFR* mutations in cancer progression.

## Overcoming Osimertinib Resistance: PDK1 Inhibitor Enhances Treatment Success

This research investigates the role of the pyruvate dehydrogenase kinase 1 in osimertinib resistance. In cancer, EGFR frequently undergoes alterations, leading to abnormal signaling that promotes cancer cell survival and proliferation. However, it’s unclear how these EGFR mutations contribute to cancer treatment resistance. The researchers, led by Wonyoung Park, discovered that a specific EGFR mutation, known as C797S, can cause resistance to osimertinib by elevating the production of a mitochondrial matrix protein called PDK1. For their investigations, they used patient samples, non-small cell lung cancer cell lines, and in vivo studies. The scientists concluded that targeting PDK1 could be a promising method for overcoming osimertinib resistance in lung cancer, potentially leading to more effective treatments for this type of cancer.

This summary was initially drafted using artificial intelligence, then revised and fact-checked by the author.

## Introduction

The epidermal growth factor receptor (EGFR), a transmembrane receptor tyrosine kinase belonging to the ErbB family, plays a crucial role in regulating cellular proliferation and development upon activation by specific ligands, such as epidermal growth factor (EGF)^1^. In tumor cells, *EGFR* frequently undergoes oncogenic alterations, resulting in aberrant signaling that, in turn, promotes cancer cell survival, invasion, and metastasis^2^.

*EGFR*-activating mutations are prevalent in non-small cell lung cancer (NSCLC), particularly in East Asia, where the incidence rate is approximately 30–40%^3^. The most frequently observed mutations in *EGFR* are the exon 19 in-frame deletion (ex19del) and the exon 21 L858R point mutation, which account for approximately 45–60% and 35–45%, respectively, of all *EGFR* mutations^4^. *EGFR*-activating mutations are associated with a poor prognosis in patients with NSCLC^5^. Although patients with NSCLC harboring *EGFR*-activating mutations exhibit high sensitivity to first-generation EGFR-tyrosine kinase inhibitors (TKIs), resistance often emerges within the first 9–14 months of therapy and is mediated primarily by the secondary T790M mutation^6^. Osimertinib, a third-generation EGFR-TKI, binds to the C797 residue in the ATP-binding site of EGFR, demonstrating significant selectivity for EGFR proteins with activating mutations or the T790M mutation^7^. Compared with those treated with first- and second-generation EGFR-TKIs, patients with *EGFR*-mutated NSCLC treated with osimertinib have prolonged overall survival^8^. Despite the significant clinical effectiveness, osimertinib therapy eventually leads to resistance, due mainly to the acquisition of the *EGFR* C797S mutation^9^. Identifying new predictive biomarkers is vital for the development of targeted therapies for lung cancer, especially therapies for overcoming resistance to drugs such as osimertinib in *EGFR*-mutated NSCLC.

Cancer cells often exhibit altered metabolic profiles that favor aerobic glycolysis rather than normal mitochondrial oxidative phosphorylation (OXPHOS) even under normoxic conditions, a phenomenon known as the Warburg effect^10^. This metabolic shift, characterized by increased glycolytic activity and lactate production, can lead to further modification of the tumor microenvironment, resulting in resistance to EGFR-TKI therapy^11^. Targeting glycolytic enzymes is a promising approach to rewire the altered tumor metabolism to sensitize (or resensitize, after resistance has developed) cancer cells to chemotherapy^12^. Pyruvate dehydrogenase kinase 1 (PDK1), a key regulator of glycolysis, is often overexpressed in cancer cells and can be activated by hypoxia-inducible factor-1 alpha (HIF-1α)^13,14^. PDK1 inhibits the pyruvate dehydrogenase (PDH) complex (PDC), preventing the conversion of pyruvate to acetyl-CoA and leading to its accumulation in the cytoplasm, where it is converted to lactate^15^. In contrast, inhibiting PDK activates PDH, which promotes the conversion of pyruvate to acetyl-CoA. This process stimulates mitochondrial respiration, resulting in the generation of excessive reactive oxygen species (ROS), which can impair mitochondrial function and activate apoptotic pathways^15^. Previous studies have demonstrated that inhibiting PDK1 can enhance the anticancer effect of EGFR-TKIs in malignant tumors, including NSCLCs^16,17^. Several studies have shown that EGFR and PDK1 interact reciprocally, promoting cancer development^18,19^. Targeting PDK1 holds promise as a therapeutic strategy for *EGFR*-mutated NSCLCs^20^. However, more research is needed, particularly in exploring the intricate association between EGFR and PDK1.

Considering these challenges, we hypothesized that targeting PDK1 could be a promising approach for overcoming osimertinib resistance in *EGFR*-mutated NSCLC. Our objective was to assess the potential increases in PDK1 expression and the level of phosphorylated pyruvate dehydrogenase E1 subunit alpha 1 (p-PDHA1) in NSCLC samples, aiming to compare prognostic outcomes to determine whether higher levels of PDK1 and p-PDHA1 are related to osimertinib resistance. The findings gained by this approach likely increase the clinical relevance established by previous studies. Finally, we sought to provide novel insights into the fundamental mechanisms contributing to anticancer drug resistance, with a particular emphasis on positioning PDK1 as a prospective molecular target for therapeutic interventions in lung cancer.

## Methods

### Antibodies and reagents

Supplementary Tables 1 and 2 provide a comprehensive list of the antibodies and reagents utilized in this study, respectively. Unless explicitly mentioned, all chemicals were procured from Sigma‒Aldrich (Burlington, MA, USA). The inhibitor concentrations and treatment durations were carefully chosen to ensure effective pathway blockade while avoiding apparent cell toxicity.

### Cell culture

A549 (ATCC, Manassas, VA, USA; CCL-185), NCI-H292 (ATCC; CRL-1848), PC-9 (ECACC, London, UK; 90071810), and NCI-H1975 (ATCC; CRL-5908) cells were cultured in RPMI 1640 medium (Welgene, Gyeongsan, Korea) supplemented with 10% heat-inactivated fetal bovine serum (FBS; Gibco, Thermo Fisher Scientific, Waltham, MA, USA) and 1% penicillin/streptomycin (Gibco). LLC (ATCC; CRL-1642) and Platinum-A (Plat-A) cells (Cell Biolabs, San Diego, CA, USA; RV-102) were cultured in Dulbecco’s modified Eagle’s medium (DMEM, Welgene) supplemented with 10% FBS and 1% penicillin/streptomycin. Plat-A cells were selected using 5 μg/mL puromycin and 10 μg/mL blasticidin to produce retroviruses. All cell lines were maintained at 37 °C with 5% CO_2_.

### CRISPR-mediated genome editing

The target sequences for *EGFR* and *PDK1* were designed using the CHOPCHOP tool (http://chopchop.cbu.uib.no) provided by the Zhang Laboratory (Cambridge, MA, USA). Supplementary Table 3 presents a list of the single guide RNAs (sgRNAs) used in this study. Complementary oligonucleotides, which contained *Bsm*BI restriction sites, were synthesized for each sgRNA (Bionics, Seoul, Korea) and subsequently cloned and inserted into the lentiCRISPR v2 vector (Addgene, Cambridge, MA, USA).

### Transient transfection

Cells (1 × 10^6^) were transfected with sgRNAs using Lipofectamine 2000 (Invitrogen, Thermo Fisher Scientific) following the manufacturer’s instructions. After 24 h of transfection, the cells were treated with 1 μg/mL puromycin. A549 *EGFR*^*L858R*+T790M+C797S^ (RMS) and A549 *EGFR*^*19del747*_750+T790M+C797S^ (19DMS) cells were cotransfected with pMX-Hygro and *PDK1* sgRNAs prior to selection using 200 μg/mL hygromycin B. *EGFR* or *PDK1* deletion in knockout cells was confirmed via western blotting.

### Isolation of *EGFR*-knockout cells using flow cytometry

A549 cells transfected with *EGFR* sgRNA1 or *EGFR* sgRNA2 were suspended in 3% bovine serum albumin (BSA) in phosphate-buffered saline at a concentration of 1 × 10^6^ cells/mL. These cells were incubated with a phycoerythrin-conjugated anti-EGFR (EGFR-PE) antibody for 30 min. Subsequently, the cells were washed and sorted based on measurements at specific excitation/emission wavelengths (488 nm/575 nm) using an automated high-speed cell sorting system (BD FACSAria III; BD Biosciences, Franklin Lakes, NJ, USA).

### Western blotting

Cells were lysed in 1% NP-40 lysis buffer containing sodium chloride (150 mM), HEPES (10 mM, pH 7.5), NP-40 (1%), sodium pyrophosphate (5 mM), sodium fluoride (5 mM), sodium orthovanadate (2 mM), and protease inhibitor cocktail (Roche Applied Science, Mannheim, Germany). Equal amounts of protein were aliquoted based on concentration measurements using the Bradford assay (Bio-Rad, CA, USA), separated using sodium dodecyl sulfate‒polyacrylamide gel electrophoresis (SDS‒PAGE), and subsequently transferred onto nitrocellulose membranes (Amersham Bioscience, Uppsala, Sweden) with a transfer system (Hoefer Inc., Holliston, MA, USA). The membranes were immersed in a blocking solution for 1 h at 24 °C to prevent the nonspecific binding of antibodies. Target protein bands were detected using an EZ-Western kit (DoGenbio, Seoul, Korea), and their densities were quantified using an ImageQuant LAS 4000 imaging system (GE Healthcare, Chicago, IL, USA).

### Construction of *EGFR* mutants

The *EGFR*^*WT*^, *EGFR*^*L858R*^, and *EGFR*^*L858R+T790M*^ plasmids were procured from Addgene (Watertown, MA, USA). The *EGFR*^*L858R+T790M+C797S*^, *EGFR*^*19del_747-750*^, *EGFR*^*19del_747-750+T790M*^, and *EGFR*^*19del_747-750+T790M+C797S*^ mutants were generated by site-directed mutagenesis using a commercial kit (iNtRON Biotechnology, Seoul, Korea) with the specific primers listed in Supplementary Table 4. The amplification reaction involved preheating the reaction mixture to 95 °C for 10 min, followed by 18 cycles of 95 °C for 30 s, 55 °C for 1 min, and 72 °C for 8 min. These mutant sequences were cloned and inserted into the pBABE vector (Addgene), and sequencing was conducted at Bionics (Seoul, Korea) to validate all mutations.

### Generation of stable cell lines via retroviral transduction

To establish stable cell lines, the pBABE-empty vector (EV) and EGFR plasmids were transfected into Plat-A cells using polyethylenimine. After 48 h of transfection, the supernatant was added to A549 EGFR^KO#1^, NCI-H292, PC-9, NCI-H1975, and LLC cells, and the cells were incubated with polybrene for 2 days. Subsequently, the transduced cells were selected by incubation with 1 μg/mL puromycin for 1 week.

### Cell viability assay

Cell viability was assessed via the 3-(4,5-dimethylthiazol-2-yl)-2,5-diphenyl-2H-tetrazolium bromide (MTT) assay. Cells were seeded in 96-well culture plates at a density of 2 ×10^4^ cells/well. The following day, the cells were treated with various drug concentrations for either 24 or 72 h. Subsequently, MTT solution (2.0 mg/mL) was added to each well, and the plates were incubated for 4 h. After removing the culture medium, the number of formazan crystals formed within the living cells was determined by measuring the absorbance at 540 nm using a SpectraMax M2 microplate reader (Molecular Devices, San Jose, CA, USA).

### Immunohistochemistry of tumor lung tissues

Immunohistochemistry (IHC) was conducted to evaluate p-PDHA1 (S293) and PDK1 protein levels in osimertinib-sensitive (OS) and osimertinib-resistant (OR) lung cancer tissues. These tissues were collected during the initial surgery from patients diagnosed with NSCLC. The procedure for preparation of lung cancer tissue sections involved the following steps: antigen retrieval, blocking of endogenous peroxidase activity, incubation with primary antibodies, subsequent incubation with horseradish peroxidase (HRP)-conjugated secondary antibodies, catalysis of the colorimetric reaction by HRP using a substrate solution, and counterstaining. Representative images were captured at magnifications of 100× and 400×, and staining patterns and distributions were subsequently analyzed.

### PDH activity assay

PDH activity was monitored using the PDH Activity Assay Kit from Sigma‒Aldrich. Cells were seeded in 6-well culture plates at a density of 5 × 10^5^ cells/well. Following cell lysis with PDH assay buffer, PDH substrates and developers were added. Enzymatic activity was subsequently determined by measuring the absorbance at 450 nm using a SpectraMax M2 microplate reader.

### 2-(7-Nitro-2,1,3-benzoxadiazol-4-yl)-d-glucosamine (2-NBDG) glucose uptake assay

Cells were seeded in 6-well culture plates at a density of 5 × 10^5^ cells/well. After pretreatment with glucose-free medium for 4 h, the cells were incubated with 2-NBDG (10 mg/mL) for 10 min at 37 °C. Cellular uptake of 2-NBDG was measured using an Attune NxT flow cytometer (Thermo Fisher Scientific) by measuring fluorescence at excitation/emission wavelengths of 485/535 nm.

### Lactate production assay

Lactate production was monitored using a lactate fluorometric assay kit from BioVision (Milpitas, CA, USA). Cells were seeded in 6-well culture plates at a density of 5 × 10^5^ cells/well. Following treatment with phenol red-free medium for 4 h, fluorescence was measured at excitation/emission wavelengths of 535/590 nm using a SpectraMax M2 microplate reader.

### Extracellular oxygen consumption assay

Extracellular oxygen consumption was measured using a commercial extracellular oxygen consumption assay kit (Abcam, Cambridge, UK) following the manufacturer’s instructions. Cells were seeded in 96-well black culture plates at a density of 1 ×10^4^ cells/well, and fluorescence (excitation/emission = 380/650 nm) was measured using a SpectraMax M2 microplate reader.

### Cell cycle analysis

Cells were seeded in 6-well plates at a density of 2 × 10^5^ cells/well. The following day, the cells were incubated with serum-free medium for 24 h. Subsequently, the cells were treated with DCA (50 mM) or leelamine (10 μM) for 15 h in serum-containing medium. After the 15 h incubation period, the cells were harvested, suspended, and fixed with 70% ethanol. Following fixation, the cells were stained with 50 μg/mL propidium iodide (Sigma‒Aldrich) in PBS supplemented with RNase A (100 μg/mL; Sigma‒Aldrich) for 30 min. The samples were analyzed using an Attune acoustic focusing cytometer (Invitrogen), and the data were analyzed using FlowJo software.

### Detection of apoptotic cells using flow cytometry

Cells were treated with various drug concentrations for 24 h, and apoptotic cells were detected using an apoptosis detection kit (BD Biosciences). Flow cytometric analysis was performed using an Attune acoustic focusing cytometer (Invitrogen), and the data were analyzed using FlowJo software.

### Colony formation assay

Cells were seeded in 6-well plates at a density of 1 × 10^3^ cells/well and incubated for 14 days. Subsequently, the cells were stained with ethidium bromide for 10 s, washed, and visualized using the Gel Doc system (Bio-Rad)^21^.

### Hematoxylin and eosin (H&E) staining

Formalin-fixed kidney and liver tissue samples from allograft models were processed to obtain thin sections. Following H&E staining, the sections were mounted on slides and examined under a light microscope at 100× magnification. All procedures were performed in accordance with safety guidelines and institutional regulations.

### Animals and tumor allograft and xenograft studies

Six-week-old male mice (C57BL/6 and BALB/c nu/nu, weighing 20–24 g, *n* = 8 mice per group) were obtained from Central Lab Animal, Inc. (Seoul, Korea). All the experimental procedures were performed in accordance with the Guidelines for the Care and Use of Laboratory Animals of the National Institutes of Health of Korea and were approved by the Institutional Animal Care and Use Committee of Pusan National University, Busan, Republic of Korea (protocol numbers: PNU-2021-0053 and PNU-2022-0241). Tumor tissue specimens were immediately harvested from the mice, and tumor volumes were measured using calipers according to the formula (length × width^2^)/2.

### Statistical analyses

**Fig. 1:**
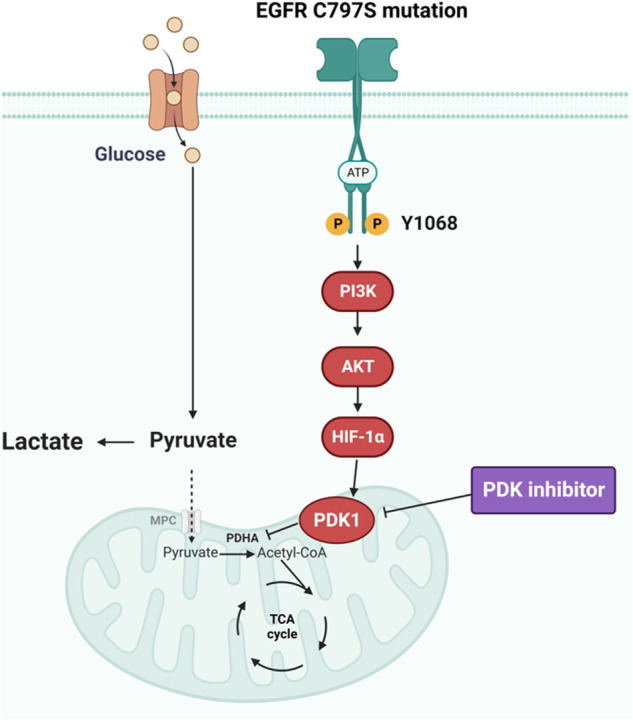
Schematic diagram showing the mechanism of osimertinib resistance driven by the *EGFR* C797S mutation. The schematic illustrates the mechanism of epidermal growth factor receptor (*EGFR*) C797S mutation-driven osimertinib resistance due to the increase in pyruvate dehydrogenase kinase (PDK)1 expression through the EGFR/Serine/Threonine Kinase 1 (AKT)/hypoxia-inducible factor (HIF)-1α axis; the combination of osimertinib with a PDK inhibitor can overcome this resistance.

The half-maximal cytotoxic concentration (CC_50_) of each drug was calculated by fitting curves in Microsoft Excel, whereas other analyses were performed using GraphPad Prism software (GraphPad Software, San Diego, CA, USA). The significance of the differences between the mean values of the two groups was analyzed using Student’s *t-*test, whereas multiple comparisons among groups were performed using a one-way analysis of variance with Dunnett’s test. The threshold for statistical significance was set at *p* < 0.05. All experiments were independently repeated three times. Figure 1 was generated with BioRender.

## Results

### OR NSCLC samples from patients exhibit increased protein levels of PDK1 and phosphorylated PDHA1

**Fig. 2:**
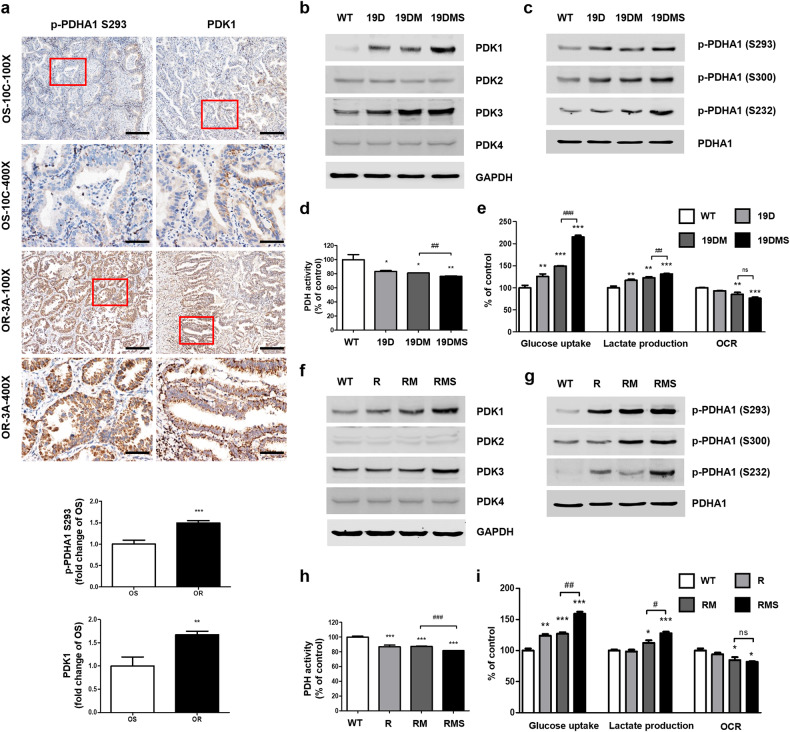
Osimertinib-resistant non-small cell lung cancers (NSCLCs) exhibit elevated PDK1 expression, followed by enhanced glycolysis. **a** Representative immunohistochemical images showing phosphorylated pyruvate dehydrogenase E1 subunit alpha 1 (p-PDHA1) (S293) and pyruvate dehydrogenase kinase 1 (PDK1) in lung cancer tissues of who are osimertinib-sensitive (OS) and osimertinib-resistant (OR) patients. The area in the red box represents a view at 400× magnification. The scale bar represents 200 μm at 100× magnification and 50 μm at 400× magnification. **b**, **f** The expression levels of PDK1, PDK2, PDK3, PDK4, and glyceraldehyde 3-phosphate dehydrogenase (GAPDH; loading control) in A549 epidermal growth factor receptor *(EGFR) wild-type* and mutant cells were measured via western blotting. **c**, **g** p-PDHA1 (S293), p-PDHA1 (S300), p-PDHA1 (S232), and total PDHA1 levels in A549 *EGFR* wild-type and mutant cells were measured via western blotting. **d**, **h** PDH activity was measured in A549 *EGFR* wild-type and mutant cells. **e**, **i** Glucose uptake, lactate production, and oxygen consumption rate (OCR) were measured in A549 *EGFR* wild-type and mutant cells. Data information: The data in **a**, **d**, **e**, **h**, and **i** are presented as the mean ± standard error of the mean (SEM) values. Statistical analysis (**a**, **d**, **e**, **h** and **i**) was conducted using Student’s *t*-test for comparisons among the OS, *EGFR*^*19del747*_750+*T790M*^ (19DM), and *EGFR*^*L858R*+*T790M*^ (RM) groups. Statistical analyses in **d**, **e**, **h**, and **i** were performed using one-way analysis of variance with Dunnett’s post hoc test, with comparison to the wild-type (WT) group. *p < 0.05, **p < 0.01, ***p < 0.001, #*p* < 0.05, ##*p* < 0.01, and ###*p* < 0.001 indicate statistically significant differences. “ns” indicates nonsignificance.

Previous studies have shown promising results using various therapeutic strategies targeting PDK1 in *EGFR*-mutant NSCLC cells^20,22,23^. However, limited clinical research has been conducted on these strategies. In this study, we investigated the association between PDK1 expression and the development of drug resistance in lung cancer tissue samples. Tumor tissues were collected from patients with NSCLC during their initial surgery, and molecular profiling was performed to identify individuals harboring *EGFR*-activating mutations (L858R and ex19del) (Supplementary Table 5). These patients were subsequently treated with first-line osimertinib. The patients were categorized into two groups based on their clinical outcomes: the OS group, comprising 10 patients who exhibited a prolonged response to osimertinib lasting 16–29 months with no severe drug-related adverse effects or signs of recurrence or metastasis; and the OR group, consisting of 10 patients who received osimertinib for a shorter duration (2–19 months) and experienced severe rash, pruritus, or metastasis, indicating acquired drug resistance. We examined the levels of p-PDHA1 (S293) and PDK1 in lung cancer tissues obtained from 20 patients with NSCLC. Immunohistochemical analysis of these lung cancer tissues revealed significantly greater levels of p-PDHA1 (S293) and PDK1 in the OR group than in the OS group (Fig. 2a and Supplementary Table 6). This finding strongly suggests a substantial correlation between elevated PDK1 expression and the development of osimertinib resistance. To further investigate this relationship, we developed additional osimertinib resistance models and conducted comprehensive experiments.

### Introduction of the C797S mutation in *EGFR* strengthens resistance to osimertinib

To gain insight into the potential mechanisms underlying osimertinib resistance, we established *EGFR*-mutant cell lines using human NSCLC A549 cells, which originally possessed wild-type *EGFR*. The CRISPR/Cas9 system was utilized to delete the endogenous *EGFR* gene (Supplementary Fig. 1), and various *EGFR* variants, including wild-type (WT) *EGFR*, *EGFR*^*19del747*_750^ (19D), *EGFR*^*19del747*_750+T790M^ (19DM), *EGFR*^*19del747*_750+T790M+C797S^ (19DMS), *EGFR*^*L858R*^ (R), *EGFR*^*L858R*+T790M^ (RM), and *EGFR*^*L858R*+T790M+C797S^ (RMS), were subsequently introduced (Supplementary Fig. 2a, b). Consistent with previous findings^24^, A549 19DMS/RMS cells demonstrated resistance to osimertinib owing to the presence of the C797S mutation (Supplementary Fig. 2c, d and Supplementary Table 7). Furthermore, osimertinib effectively suppressed EGFR downstream signaling pathways, including both the phosphatidylinositol-3 kinase (PI3K)/AKT and mitogen-activated protein kinase (MAPK)/extracellular signal-regulated kinase (ERK) cascades, in *EGFR* WT, single-mutant, and double-mutant A549 cells (Supplementary Fig. 2e, f). Conversely, in triple-mutant cells harboring C797S, we observed sustained EGFR phosphorylation, resulting in a compromised ability of osimertinib to inhibit AKT and ERK signaling. Notably, the reduced impact of osimertinib on the Janus kinase (JAK)/signal transducer and activator of transcription 3 (STAT3) pathway across all cell lines aligned with previously reported findings^25^.

To explore resistance mechanisms in lung cancer cell lines other than A549, we constructed two series of *EGFR* mutants, namely, *EGFR* double mutants (19DM or RM) and C797S-containing *EGFR* triple mutants (19DMS or RMS), in human lung mucoepidermoid carcinoma H292 cells as well as human NSCLC PC-9 and H1975 cells (Supplementary Fig. 3a, c, e, and g). These respective cell lines harbor WT *EGFR*, *EGFR* with exon 19 deletion, and *EGFR* with a double mutation (L858R and T790M). Regardless of the baseline mutations, the presence of the C797S-containing *EGFR* triple mutant consistently resulted in osimertinib resistance (Supplementary Fig. 3b, d, f, h, and Supplementary Table 8).

These findings strongly implicate the C797S mutation in *EGFR* as the primary driver of osimertinib resistance in various NSCLC cell lines. Next, we aimed to further explore the mechanisms connecting PDK1 to osimertinib resistance.

### The *EGFR* C797S mutation promotes elevated PDK1 expression followed by enhanced glycolysis

To establish an association between the increased PDK1 expression observed in patient tissue samples and C797S-induced osimertinib resistance, we analyzed PDK expression in A549 cells harboring WT or mutant *EGFR*. Among the four PDKs examined, only PDK1 was upregulated in response to the introduction of the C797S mutation (Fig. 2b, f). Conversely, the introduction of the C797S mutation did not lead to a significant increase in PDK3 expression. PDK2 and PDK4 maintained consistent expression levels across all genotypes. Concurrently, we observed increases in the phosphorylation of PDHA1 at S293, S300, and S232 (Fig. 2c, g). These increases were accompanied by reduced PDH activity in A549 19DMS/RMS cells (Fig. 2d, h). While all four PDKs phosphorylate S293 and S300 of PDHA1, S232 is phosphorylated exclusively by PDK1^26^. These findings align with the elevated PDK1 expression observed in patients with osimertinib resistance. Furthermore, compared with *EGFR*-WT cells, EGFR-mutant cells, particularly A549 19DMS/RMS cells, displayed increased glucose uptake and lactate production. Conversely, the oxygen consumption rate (OCR) decreased in A549 19DMS/RMS cells (Fig. 2e, i). Osimertinib treatment did not induce significant changes in glucose uptake, lactate production, or the OCR in A549 19DMS/RMS cells (Supplementary Fig. 4).

Similar alterations in glycolysis were observed in H292, PC9, and H1975 cells carrying the *EGFR* C797S mutation: (1) an increase in PDK1 expression without a significant change in the expression of PDK3, (2) an elevated p-PDHA1 (S293) level along with reduced PDH activity, and (3) increased glucose uptake and lactate production accompanied by a decreased OCR (Supplementary Fig. 5). Collectively, these findings indicate that the acquired *EGFR* C797S mutation leads to increased PDK1 expression, resulting in enhanced glycolysis and reduced OXPHOS.

To explore the therapeutic potential of targeting PDK1, we assessed the sensitivity of *EGFR*-mutant cells to two PDK1 inhibitors, dichloroacetate (DCA) and leelamine. Considering that glycolysis-dependent cancer cells are more susceptible to PDK1 inhibition, leading to increased cellular oxidative stress and apoptosis^27^, we hypothesized that *EGFR* C797S mutant cells, characterized by elevated glycolytic activity relative to that in *EGFR* double-mutant cells, would exhibit increased susceptibility to PDK1 inhibitors. Concordantly, our experiments revealed that *EGFR* C797S mutant cells displayed significantly greater sensitivity to both DCA and leelamine than did *EGFR* double-mutant cells (Supplementary Fig. 6 and Supplementary Tables 9 and 10). We also found that treatment with DCA or leelamine did not significantly alter cell proliferation or induce cell cycle arrest (Supplementary Fig. 7).

These findings validate the hypothesis that the *EGFR* C797S mutation causes metabolic changes, highlighting the potential therapeutic utility of targeting PDK1 to overcome osimertinib resistance in *EGFR*-mutant lung cancer cells.

### PDK1 upregulation in *EGFR* C797S mutant cells is mediated through the EGFR/AKT/HIF-1α axis

**Fig. 3:**
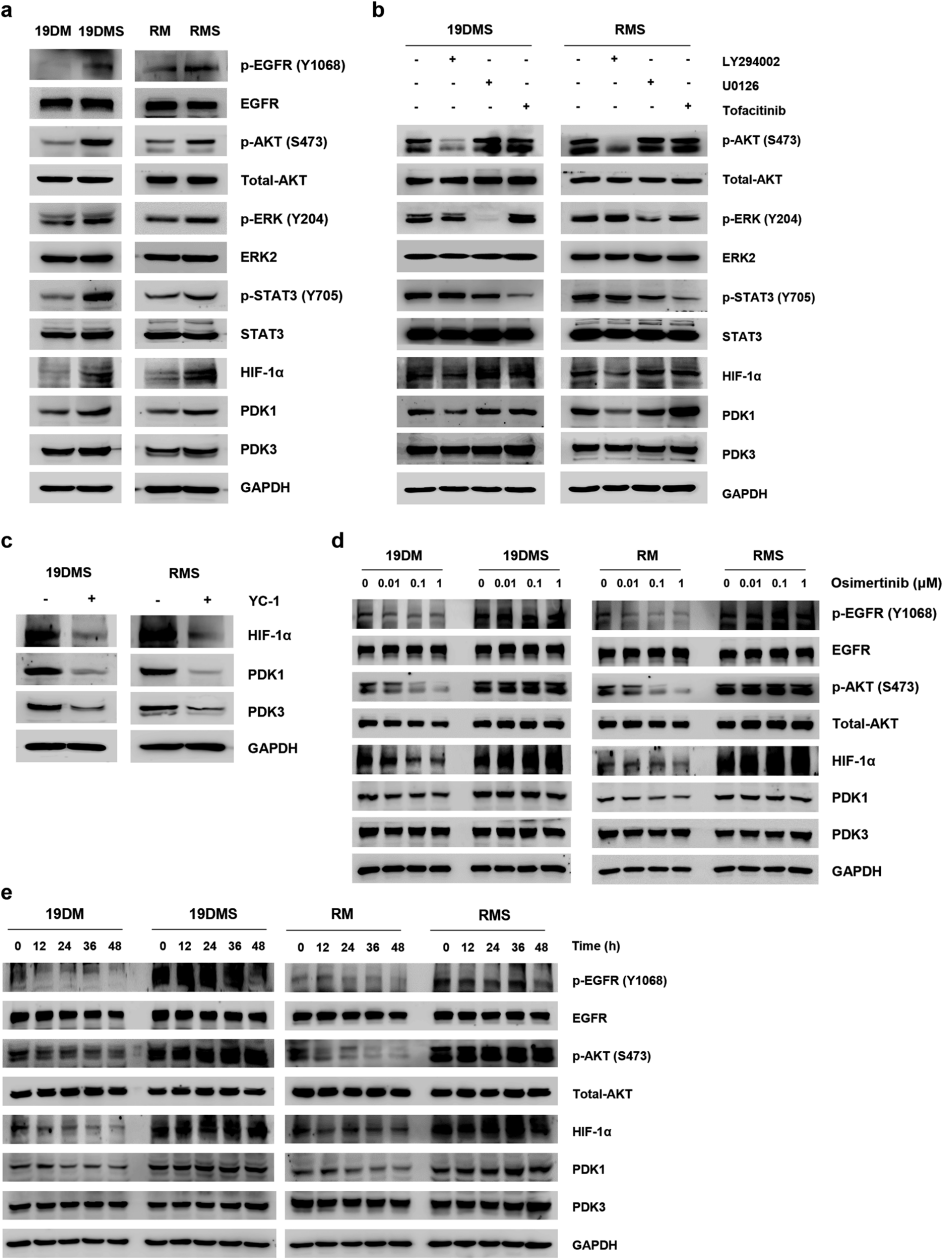
The epidermal growth factor receptor *(EGFR)* C797S mutation results in PDK1 upregulation via the EGFR/AKT/HIF-1α axis in A549 cells. **a** Immunoblot analysis results showing the levels of p-EGFR (Y1068), p-AKT Serine/Threonine Kinase 1 (AKT) (S473), p-extracellular signal-regulated kinase (ERK) (Y204), and p-signal transducer and activator of transcription 3 (STAT3) (Y705), as well as the levels of EGFR, total AKT, ERK2, STAT3, hypoxia-inducible factor (HIF)-1α, pyruvate dehydrogenase kinase (PDK)1, PDK3, and glyceraldehyde 3-phosphate dehydrogenase (GAPDH; loading control) in *EGFR*-mutant A549 cells. **b** Immunoblot analysis of A549 *EGFR*^*19del747*_750+*T790M+C797S*^ (19DMS)/*EGFR*^*L858R*+*T790M+C797S*^ (RMS) cells treated with LY294002 (50 μM), U0126 (10 μM), or tofacitinib (50 μM) for 24 h. The levels of p-AKT (S473), p-ERK (Y204), and p-STAT3 (Y705), as well as the levels of total AKT, ERK2, STAT3, HIF-1α, PDK1, PDK3, and GAPDH (loading control), were measured. **c** Immunoblot analysis of A549 19DMS/RMS cells treated with YC-1 (50 μM) for 24 h. The expression of HIF-1α, PDK1, PDK3, and GAPDH (loading control) was measured. **d** Immunoblot analysis of A549 19DMS/RMS cells treated with the indicated concentrations of osimertinib for 24 h. The levels of p-EGFR (Y1068) and p-AKT (S473), as well as the levels of EGFR, total AKT, HIF-1α, PDK1, PDK3, and GAPDH (loading control), were measured. **e** Immunoblot analysis of A549 19DMS/RMS cells treated with osimertinib (0.1 μM) at the indicated time points. The levels of p-EGFR (Y1068) and p-AKT (S473), as well as the levels of EGFR, total AKT, HIF-1α, PDK1, PDK3, and GAPDH (loading control), were measured.

To elucidate the mechanisms by which the *EGFR* C797S mutation results in upregulation of PDK1 expression, thereby contributing to osimertinib resistance, we assessed the basal phosphorylation level of EGFR in *EGFR* double- and triple-mutant cells. Notably, we observed increased EGFR phosphorylation even in the absence of EGF, which led to the activation of downstream signaling pathways, as indicated by the increases in the p-AKT, p-ERK, and p-STAT3 levels (Fig. 3a and Supplementary Fig. 8a, b). Considering that the activity of oncogenic signaling pathways can induce the expression of HIF-1α^28^, we investigated the role of HIF-1α in *EGFR* C797S mutant cells. Consistent with prior research, we detected an elevated level of HIF-1α in *EGFR* C797S mutant cells (Fig. 3a and Supplementary Fig. 8a, b).

To further investigate the sequential events and molecular interactions, as well as their consequent effects, we employed pharmacological interventions, including LY294002 (a PI3K-specific inhibitor), U0126 (a MEK-targeting inhibitor), tofacitinib (a JAK signaling inhibitor), and YC-1 (a potent HIF-1α inhibitor). In A549 19DMS/RMS cells, LY29004 effectively suppressed the expression of HIF-1α and PDK1, whereas YC-1 effectively downregulated the expression of both PDK1 and PDK3 (Fig. 3b, c). This activation of oncogenic signaling pathways significantly contributed to increased cell proliferation, as was evident in both in vitro and xenograft models established with *EGFR* C797S mutant cells (Supplementary Fig. 9).

Subsequently, we examined the effects of osimertinib treatment on downstream signaling pathways. Notably, even at 1 μM, osimertinib failed to decrease the levels of activated proteins, including p-EGFR, p-AKT, HIF-1α, and PDK1, in *EGFR* C797S mutant cells (Fig. 3d and Supplementary Fig. 8c, d). Conversely, in A549 RM/19DM cells, a considerably lower concentration of osimertinib effectively and time-dependently reduced the levels of p-EGFR, p-AKT, HIF-1α, and PDK1 (Fig. 2e). These findings indicate that the upregulation of PDK1 in *EGFR* C797S mutant cells is mediated primarily via the EGFR/AKT/HIF-1α signaling axis.

### PDK inhibitors synergistically enhance osimertinib-induced apoptosis in *EGFR* C797S mutant cells

Our comprehensive investigation of *EGFR* C797S mutation-mediated osimertinib resistance led us to explore the upstream signaling pathways, including the EGFR, AKT, and HIF-1α pathways. These investigations revealed substantial upregulation of the EGFR/AKT/HIF-1α axis, resulting in elevated expression of PDK1, a critical contributor to osimertinib resistance.

**Fig. 4:**
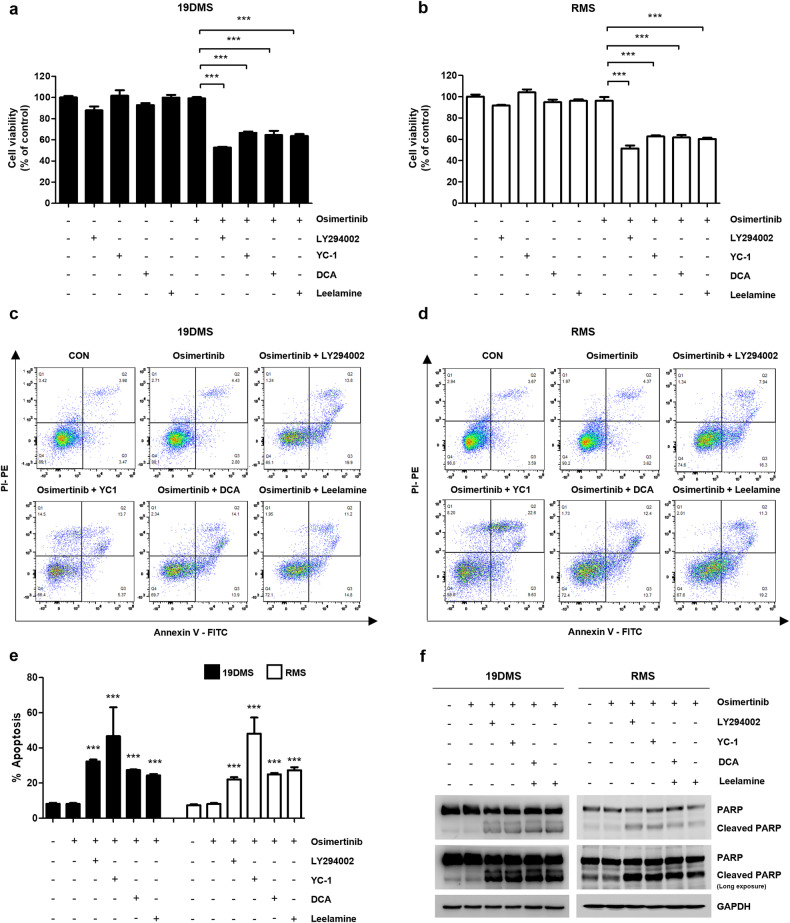
The combination of pyruvate dehydrogenase kinase (PDK) inhibitors and osimertinib amplifies apoptosis in A549 epidermal growth factor receptor *(EGFR)* C797S mutant cells. A549 *EGFR*^*19del747*_750+*T790M+C797S*^ (19DMS)/*EGFR*^*L858R*+*T790M+C797S*^ (RMS) cells were treated with osimertinib (1 μM) alone or in combination with LY294002 (50 μM), YC-1 (50 μM), DCA (15 mM), or leelamine (3 μM) for 24 h. **a**, **b** Cell viability was assessed using the 3-(4,5-dimethylthiazol-2-yl)-2,5-diphenyl-2H-tetrazolium bromide (MTT) assay. **c-e** Apoptotic cells were detected using Annexin V/PI staining and flow cytometry; flow cytometry-based dot plots of the Annexin V/PI assay are shown. **f** Western blotting was performed to evaluate the levels of poly (ADP-ribose) polymerase (PARP), cleaved PARP, and glyceraldehyde 3-phosphate dehydrogenase (GAPDH; loading control). Data information: The data in **a**, **b**, and **e** are presented as the mean ± standard error of the mean (SEM) values. Statistical analyses in **a** and **b** were conducted using a one-way analysis of variance with Dunnett’s post hoc test, with comparison to the osimertinib treatment group (sixth lane). Statistical analysis in **e** was performed using one-way analysis of variance with Dunnett’s post hoc test, with comparison to the control group (the first lane for 19DMS or RMS cells). ***p < 0.001.

Building upon these findings, we explored the therapeutic implications of targeting this signaling axis. Notably, when osimertinib was combined with LY29004 or YC-1, a significant increase in cell death was observed (Fig. 4a, b). This combination strategy effectively disrupted the PI3K/AKT/HIF-1α signaling axis, as evidenced by its synergistic effect. Furthermore, the addition of these inhibitors restored the ability of osimertinib to induce the cleavage of poly (ADP-ribose) polymerase (PARP), a key hallmark of apoptosis, and triggered apoptosis in A549 19DMS/RMS cells (Fig. 4c–f).

Subsequently, we focused on the specific targeting of PDK1, a downstream activator of the EGFR/AKT/HIF-1α activation cascade. We used two PDK1 inhibitors, DCA and leelamine, in combination with osimertinib and observed that compared to the upstream inhibitors, these combinations induced similar levels of apoptosis (Fig. 4). Notably, these synergistic effects were consistently replicated in H292 19DMS/RMS cells, PC9 19DMS cells, and H1975 RMS cells when osimertinib was combined with a PI3K inhibitor or a HIF-1α inhibitor, both of which target the PI3K/AKT/HIF-1α signaling pathway, or with a PDK1 inhibitor, which acts downstream of EGFR/AKT/HIF-1α activation (Supplementary Fig. 10).

These results highlight the potential of targeting PDK1 as a promising therapeutic approach for overcoming *EGFR* C797S-mediated osimertinib resistance, opening new avenues for the effective treatment of drug-resistant NSCLC.

### Targeting PDK1 cooperatively enhances osimertinib sensitivity in *EGFR* C797S mutant cells

**Fig. 5:**
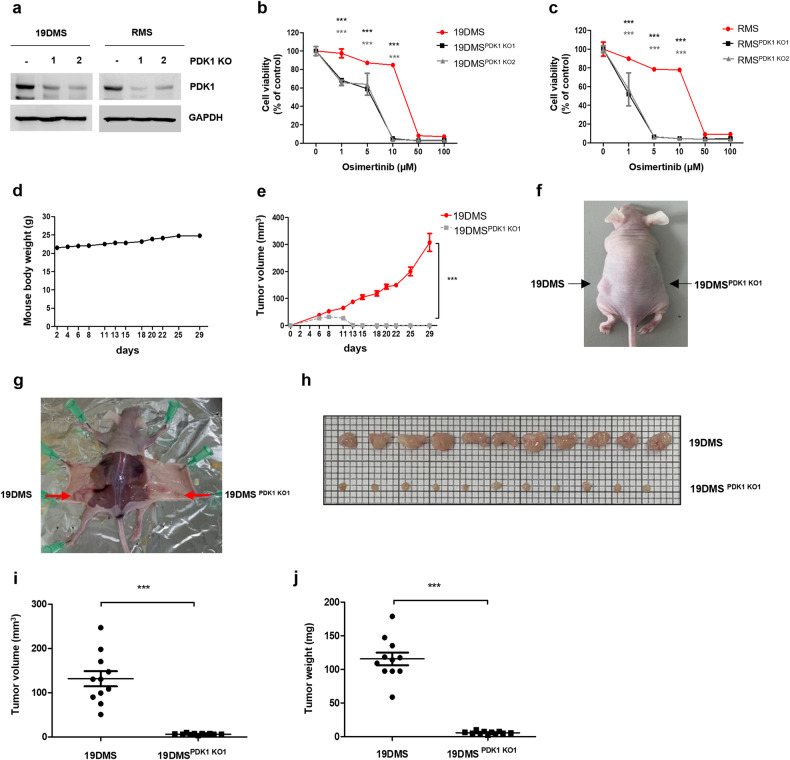
Combined targeting of PDK1 inhibits xenograft tumor formation and increases osimertinib sensitivity in A549 *EGFR* C797S mutant cells. **a** Pyruvate dehydrogenase kinase (*PDK*)1 sgRNA was transfected into A549 *EGFR*^*19del747*_750+*T790M+C797S*^ (19DMS)/*EGFR*^*L858R*+*T790M+C797S*^ (RMS) cells, and PDK1 and glyceraldehyde 3-phosphate dehydrogenase (GAPDH; loading control) expression was measured by immunoblotting. **b** Treatment of A549 *EGFR*^*19del747*_750+*T790M+C797S*^ (19DMS), 19DMS^PDK1 KO1^, and 19DMS^PDK1 KO2^ cells with osimertinib for 72 h, followed by measurement of cell viability using the 3-(4,5-dimethylthiazol-2-yl)-2,5-diphenyl-2H-tetrazolium bromide (MTT) assay. **c** Treatment of A549 *EGFR*^*L858R*+*T790M+C797S*^ (RMS), RMS^PDK1 KO1^, and RMS^PDK1 KO2^ cells with osimertinib for 72 h, followed by the measurement of cell viability using the MTT assay. **d**–**j** A549 19DMS (left) or A549 19DMS^PDK1 KO1^ (right) cells (1 × 10^7^) suspended in 100 μL of sterile phosphate-buffered saline were injected subcutaneously into BALB/c nude mice. Mouse body weight (**d**) and tumor volume (**e**) were monitored. Representative images of mice (**f**, **g**) and tumors (**h**). Tumor volume (**i**) and tumor weight (**j**). Data information: The data in **b**, **c**, **e**, **i**, and **j** are presented as the mean ± standard error of the mean (SEM) values. Statistical analyses in **b**, **c** were conducted using Student’s *t*-test, with comparison to the control group (**b**, 19DMS; **c**, RMS). Statistical analyses in **e**, **i**, and **j** were performed using Student’s *t*-test, with comparison to the 19DMS group. ***p < 0.001.

To further investigate the effect of PDK1 inhibition on *EGFR* C797S mutant cells, we used CRISPR/Cas9 gene editing to knock out *PDK1* in A549 19DMS/RMS cells (Fig. 5a). *PDK1* knockout cells exhibited increased sensitivity to osimertinib (Fig. 5b, c and Supplementary Table 11). Additionally, *PDK1* knockout reduced the proliferative capacity of A549 19DMS/RMS cells (Supplementary Fig. 11). In the xenograft model, tumors formed from A549 19DMS^*PDK1 KO1*^ cells exhibited significantly slower growth than did those formed from A549 19DMS cells injected into nude mice (Fig. 5d–j). These findings underscore the potential of PDK1 inhibition as a viable strategy for enhancing the efficacy of osimertinib and attenuating the tumorigenic capacity of *EGFR* C797S mutant cells.

### Combination treatment with osimertinib and leelamine successfully suppresses *EGFR*-mutant tumor growth in vivo

**Fig. 6:**
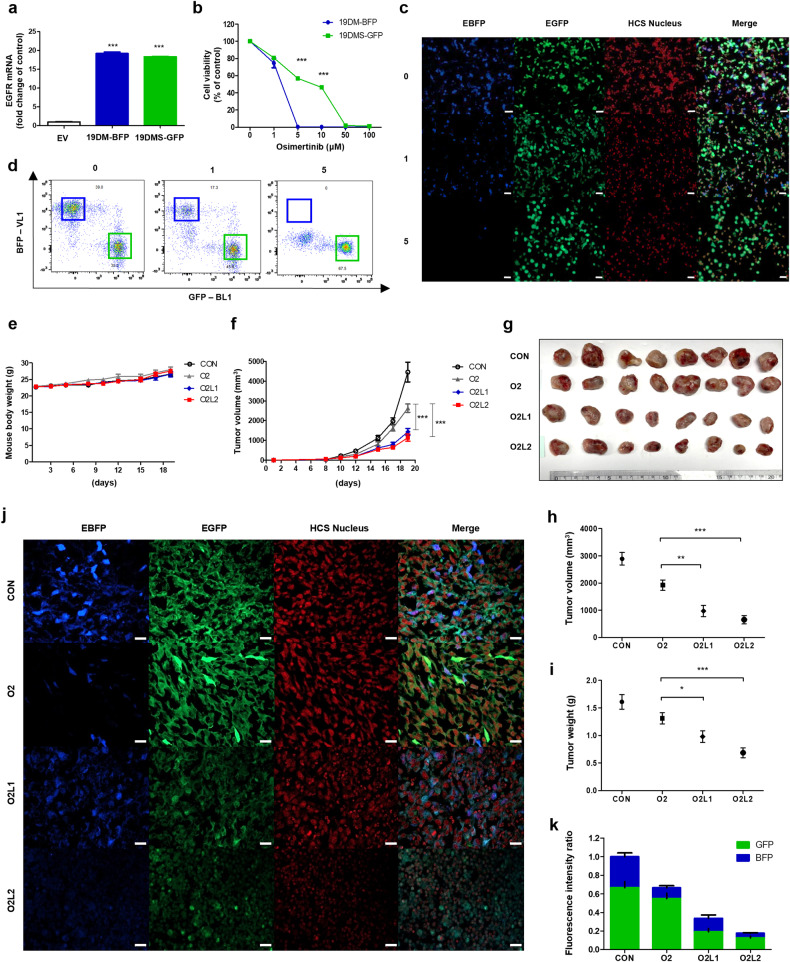
The combination of osimertinib and leelamine effectively inhibits *EGFR* C797S mutant tumor growth in vivo. **a** Confirmation of epidermal growth factor receptor (*EGFR*) mutant (*EGFR*^*19del747*_750+*T790M*^ (19DM)-blue fluorescent protein (BFP)) or *EGFR*^*19del747*_750+*T790M+C797S*^ (19DMS)-green fluorescent protein (GFP) overexpression in LLC cells using qPCR analysis of *EGFR* mRNA expression. **b** Assessment of the cytotoxic effects of osimertinib on *EGFR*-mutant LLC cells (19DM-BFP or 19DMS-GFP) using the 3-(4,5-dimethylthiazol-2-yl)-2,5-diphenyl-2H-tetrazolium bromide (MTT) assay after treatment with the indicated concentrations of osimertinib for 72 h. **c** Fluorescence microscopy images showing a 1:1 mixture of *EGFR*-mutant 19DM-BFP and 19DMS-GFP cells treated with osimertinib (0, 1, 5 μM) for 72 h. The scale bar represents 50 μm. **d** Flow cytometry data confirming the ratio of BFP to GFP in LLC 19DM-BFP and LLC 19DMS-GFP cells mixed at a 1:1 ratio and treated with osimertinib (0, 1, 5 μM) for 72 h. **e**–**k** Allograft models were established by injecting a 1:1 mixture of 19DM-BFP and LLC 19DMS-GFP LLC cells into C57BL/6 mice. After one week, the mice were treated with corn oil (CON), 2 mg/kg osimertinib (O2), 2 mg/kg osimertinib + 1 mg/kg leelamine (O2L1), or 2 mg/kg osimertinib + 2 mg/kg leelamine (O2L2) for two weeks. Body weight (**e**) and tumor volume (**f**) were measured during the treatment period. The mice were euthanized, tumor samples were harvested, and the tumors were imaged (**g**). Tumor volume (**h**) and weight (**i**) were also analyzed. Imaging of the tumors was performed using a confocal microscope (**j**). The scale bar represents 20 μm. The fluorescence intensity ratio of 19DM-BFP and 19DMS-GFP LLC cells was quantified using ImageJ software, and the bar length indicates the relative tumor volume across different treatment groups (**k**). Data information: The data in **b**, **f**, **h**, and **i** are presented as the mean ± standard error of the mean (SEM) values. Statistical analysis in **b** was conducted using Student’s *t*-test, with comparison to the 19DM group. Statistical analyses in **f**, **h**, and **i** were performed using a one-way analysis of variance with Dunnett’s post hoc test, with comparison to the O2 group. *p < 0.05, **p < 0.01, and ***p < 0.001.

Considering the promising results of combined treatment with osimertinib and leelamine in cell-based experiments, we assessed the efficacy of this combination therapy in an in vivo animal model using LLC cells harboring mutant *EGFR* (Fig. 6a). Initially, we examined the cytotoxic effect of osimertinib on LLC cells with acquired resistance owing to the *EGFR* C797S mutation (Fig. 6b). To simulate the tumor heterogeneity encountered in clinical settings, we cocultured LLC 19DM and LLC 19DMS cells and evaluated their response to osimertinib treatment using fluorescence microscopy (Fig. 6c). Flow cytometric analysis confirmed that osimertinib alone did not induce the death of *EGFR* C797S mutant cells, as evidenced by the altered ratio of blue fluorescent protein (BFP) and green fluorescent protein (GFP) signals (Fig. 6d).

In the in vivo study, the combination of osimertinib and leelamine significantly reduced tumor growth compared with that in the group treated with osimertinib alone (Fig. 6e–g). After the mice were euthanized, both the tumor volume (Fig. 6h) and tumor weight (Fig. 6i) were found to be significantly decreased with the addition of leelamine to osimertinib treatment. Notably, this reduction in tumor size was achieved without any renal or hepatic cytotoxicity, as evidenced by the absence of histopathological alterations in these organs (Supplementary Fig. 12). Conversely, osimertinib monotherapy failed to induce the death of LLC cells with the *EGFR* C797S mutation, highlighting its limited impact on *EGFR* triple-mutant cells (Fig. 6j). However, when osimertinib was combined with leelamine, cancer cell death was observed, particularly in the O2L2 group, where osimertinib was administered in combination with leelamine at a dose of 2 mg/kg (Fig. 6k).

Collectively, these findings strongly suggest that the combination of osimertinib and leelamine is a promising therapeutic approach for *EGFR*-mutant cancers, particularly those with the challenging C797S mutation, which confers resistance to osimertinib.

## Discussion

Favorable outcomes have been observed when DCA is combined with first-generation EGFR-TKIs such as erlotinib and gefitinib^22^. Additionally, the potential of pairing DCA with rociletinib, a third-generation EGFR-TKI, in combination with radiation therapy has been demonstrated as a promising therapeutic strategy in NSCLC cell line models in vitro^20^. However, these findings are primarily limited to in vitro experiments and lack essential clinical data for validation. We initially explored the mechanisms contributing to osimertinib resistance and observed a substantial increase in PDK1 levels in patients with NSCLC who exhibited osimertinib resistance (Fig. 2a). However, we cannot rule out the possibility of multiple resistance mechanisms in addition to the *EGFR* C797S mutation in OR patients. However, we strategically focused our in vitro cell line model on the *EGFR* C797S mutation owing to its prominent role as the primary driver of acquired osimertinib resistance. This mutation is the most prevalent among the known triple mutations and significantly contributes to the osimertinib resistance mechanism^9^. Further research is required to clarify the associations among osimertinib resistance, elevated PDK1 expression, and the *EGFR* C797S mutation in patients.

We further simulated common clinical scenarios observed in patients with NSCLC. Patients often present with *EGFR*-activating mutations, such as exon 19 deletion or the L858R point mutation in the receptor tyrosine kinase domain, and first-generation EGFR-TKIs are prescribed as first-line treatment for these patients^29^. Subsequently, some patients acquire resistance through various mechanisms after 9–14 months of treatment with first- or second-generation EGFR-TKIs. The T790M mutation in *EGFR* exon 20 is the predominant cause, necessitating the use of third-generation EGFR-TKIs^30^. However, patients with lung cancer eventually become resistant to even third-generation EGFR-TKIs within a few months through the acquisition of the C797S mutation, which is located within the tyrosine kinase domain^24^. Our findings are relevant because the acquisition of the C797S mutation can further complicate the treatment landscape. To comprehensively model clinical progression, we established various cell lines with different combinations of *EGFR* mutations, including single, double, and triple mutations. Notably, our study extended beyond these mutations to explore the specific impact of the T790M + C797S double mutation and revealed important insights. Our findings indicate that when *EGFR* carries only the T790M + C797S double mutation, without exon 19 deletion or the L858R point mutation, resistance to osimertinib is less pronounced (Supplementary Fig. 13). These findings suggest that certain primary mutations, such as exon 19 deletion or L858R, play a pivotal role in determining the degree of osimertinib resistance.

Tyrosine 1068 (Y1068) phosphorylation of EGFR is crucial for the activation of downstream signaling pathways through the interactions of EGFR with growth factor receptor-bound protein 2 (Grb2) or Grb2-associated binder 1^31^ and serves as a predictive biomarker for assessing clinical responses to EGFR-TKIs^32^. Both exon 19 deletion and the L858R point mutation in *EGFR* reportedly increase EGFR phosphorylation at Y1068, resulting in sustained activation of the EGFR signaling pathway^33^. L858, located within the activation loop of EGFR, maintains EGFR in its inactive conformation by engaging in essential hydrophobic interactions with residues in the N-lobe^34^. However, the L858R mutation, which results in the substitution of a smaller amino acid (leucine) with a larger amino acid (arginine), disrupts this inactive conformation, resulting in constitutive EGFR activation^35^. Previous studies have suggested that such alterations in amino acid properties may lead to changes in protein‒protein interactions^36^. This shift from a hydrophobic to a charged residue could impact the structural dynamics of the protein and its interactions with other cellular components. Similarly, *EGFR* exon 19 deletion disrupts the inactive conformation by shortening the β3-αC loop, which typically prevents the outward rotation of the αC-helix^37^. Furthermore, the *EGFR* T790M mutation increases EGFR phosphorylation at Y1068^38^. Our findings confirm that the acquired *EGFR* C797S mutation increases Y1068 phosphorylation and activates downstream signaling pathways without requiring external activation by growth factors such as EGF (Fig. 3a and Supplementary Fig. 8a, b). The C797S mutation may further complicate the protein structure and activate EGFR. However, further structural studies are necessary to comprehensively elucidate these phenomena.

Excessive activation of EGFR signaling pathways has been linked to inhibition of apoptosis and promotion of tumor growth^39^. The PI3K/AKT pathway is the primary downstream signaling pathway activated by EGFR^40^. The phosphorylated tyrosine kinase domain of EGFR creates a binding site for PI3K, which then induces the production of phosphatidylinositol-3,4,5-triphosphate (PIP-3), subsequently leading to AKT activation^41^. Recent research has highlighted the critical role of PI3K/AKT signaling in regulating and inducing the activation of HIF-1α across various human cancer types^42^. HIF-1α functions as a transcription factor that governs cellular responses to low oxygen levels and actively suppresses the TCA cycle by directly activating the genes encoding PDK1 and PDK3^43,44^. The PDK family comprises four isoforms, i.e., PDK1–PDK4, among which PDK-1 and PDK-3 are typically overexpressed in cancers, whereas PDK-2 and PDK-4 are expressed predominantly in tissues with high metabolic activity, such as skeletal and heart muscle^45^. In this study, we investigated the impact of the *EGFR* C797S mutation on PDK1 expression. We observed a substantial increase in PDK1 expression in *EGFR* C797S mutant cells, which was mediated primarily through the EGFR/AKT/HIF-1α axis (Figs. 2 and 3). This mutation seems to result in PDK1 upregulation, potentially contributing to the altered metabolism observed in cancer cells. In particular, analysis of the GSE19188 dataset revealed significantly elevated PDK1 levels in NSCLC tumor tissues compared with normal tissues^14^. Furthermore, we observed increased PDK1 expression in OR patients with NSCLC, as well as significant upregulation of PDK1 in *EGFR* C797S mutant cells. Collectively, these findings indicate that elevated PDK1 levels are associated with cancer progression and osimertinib resistance mechanisms.

Our study explored combination therapies to overcome osimertinib resistance in EGFR-mutant NSCLC. By targeting PDK1 with agents such as LY294002, YC-1, leelamine, and DCA in combination with osimertinib, we demonstrated a synergistic approach for sensitizing cancer cells to osimertinib. Recent studies on cancer treatment have shifted away from single-drug approaches to combination therapy strategies^46^. Combination therapy offers the advantages of enhanced treatment efficacy, a lower dosage requirement, a reduction in side effects, and a comprehensive approach to cancer treatment by leveraging synergistic interactions between different treatment approaches^47^. The sensitivity of A549 19DMS cells to osimertinib increased approximately 105-fold and that of A549 RMS cells increased approximately 155-fold when leelamine (1 μM) was combined with osimertinib (Supplementary Fig. 14 and Supplementary Table 12). The reduction in cell viability and induction of apoptosis, particularly in the presence of osimertinib, underscore the synergistic potential of these combinations in overcoming resistance, with the use of subcytotoxic doses in an effort to optimize therapeutic benefit and minimize toxicity, emphasizing the importance of tailored combination therapies in addressing cancer resistance mechanisms and guiding future clinical studies to improve patient outcomes.

PDK inhibitors have demonstrated promise in activating PDC, shifting metabolism toward OXPHOS, and promoting apoptosis in cancer cells^15,48,49^. However, despite its selectivity for *EGFR* C797S cells, DCA, a well-known PDK inhibitor, has limitations owing to its high dose requirement (millimolar concentrations) (Supplementary Fig. 6 and Supplementary Table 9). To address the need for a more specific PDK inhibitor, we developed a cell-based approach that can be used to distinguish the activity of different PDK inhibitors. In this system, leelamine was identified as exhibiting substantial selectivity^50^. Notably, leelamine had significant cytotoxic effects on *EGFR* triple-mutant cells, as evidenced by its CC_50_ values ranging from 0.01–6.78 µM (Supplementary Table 10). Previous research revealed the antitumor efficacy of leelamine at a dose of 7.5–80 mg/kg^51^. In this study, we administered leelamine at lower doses (1 or 2 mg/kg) in combination with osimertinib, and the combination effectively inhibited tumor growth (Fig. 5). This suggests the potential of combining osimertinib and leelamine to effectively target diverse cell populations within the heterogeneous tumor microenvironment. These findings provide a strong foundation for further exploration of the synergistic effects of osimertinib and leelamine as promising therapeutic agents.

This study has several limitations. First, it relies heavily on in vitro cell line models, which may not fully reproduce the complex properties of tumors under physiological conditions in humans. Although we detected distinct regulatory patterns of PDK1 and PDK3 in cell lines with different *EGFR* mutation profiles, the specific underlying reasons for these differences remain unknown. Additionally, the clinical sample size of this study was limited. Our primary focus was on the influence of the *EGFR* C797S mutation; thus, our study potentially oversimplified osimertinib resistance by not extensively exploring other resistance mechanisms and *EGFR* mutations. According to the existing NCCN guidelines^52^, detection of the *EGFR* C797S mutation is not currently a part of routine clinical evaluations, which is another limitation of our clinical dataset. Therefore, we did not specifically determine whether patients in the OR group had the *EGFR* C797S mutation. Furthermore, we did not comprehensively assess the long-term effects and potential toxicity of leelamine.

Despite these limitations, this study has several strengths. The observed increase in PDK1 expression in OR patients suggests the potential efficacy of targeting PDK1 to overcome C797S mutation-mediated resistance and implies broader applications across various osimertinib resistance mechanisms. To bolster the robustness of this study, we plan to increase the clinical sample size in our future research. By investigating novel biomarkers such as PDK1 and exploring the potential synergistic effects of PDK inhibitors, our findings offer a promising approach for further investigation with significant clinical implications.

In conclusion, we found that the *EGFR* C797S mutation increases glycolytic activity via PDK1 upregulation, leading to decreased sensitivity to osimertinib. We demonstrated that targeting PDK1 or the EGFR/AKT/HIF-1α pathway can overcome this resistance and trigger apoptosis. Furthermore, the combination of osimertinib and PDK inhibitors demonstrated promising outcomes in preclinical models. These findings provide valuable insights into the mechanisms underlying *EGFR* C797S mutation-mediated osimertinib resistance and suggest potential therapeutic approaches for patients with NSCLC.

### Supplementary information

Supplementary Materials

## Data Availability

All the data will be made available upon reasonable request.
