# Supplementary material for: Targeting pyruvate dehydrogenase kinase 1 overcomes EGFR C797S mutation-driven osimertinib resistance in non-small cell lung cancer
Source: Exp Mol Med. 2024 May 1;56(5):1137–49. doi: 10.1038/s12276-024-01221-2 (PMC11148081; doi:10.1038/s12276-024-01221-2)
Supplement: Supplementary file 1 — Supplementary Materials [file 12276_2024_1221_MOESM1_ESM.pdf]

Supplementary Materials

Targeting pyruvate dehydrogenase kinase 1 overcomes *EGFR* C797S mutation-driven osimertinib resistance in non-small cell lung cancer

Wonyoung Park[1,2], Shibo Wei[3], Chu-Long Xie[4,5], Jung Ho Han[6], Bo-Sung Kim[1,2], Bosung Kim[1,2], Jung-Sook Jin[2], Eun-Sun Yang[2], Min Kyoung Cho[7], Dongryeol Ryu[8], Hao-Xian Yang[4,5], Sung-Jin Bae[7*], Ki-Tae Ha[1,2*]

Correspondence to: Dr.BaeSJ@kosin.ac.kr; hagis@pusan.ac.kr

This PDF file includes:

Supplementary Fig. 1–14 with their legends

Supplementary Tables 1–12

Supplementary Figures

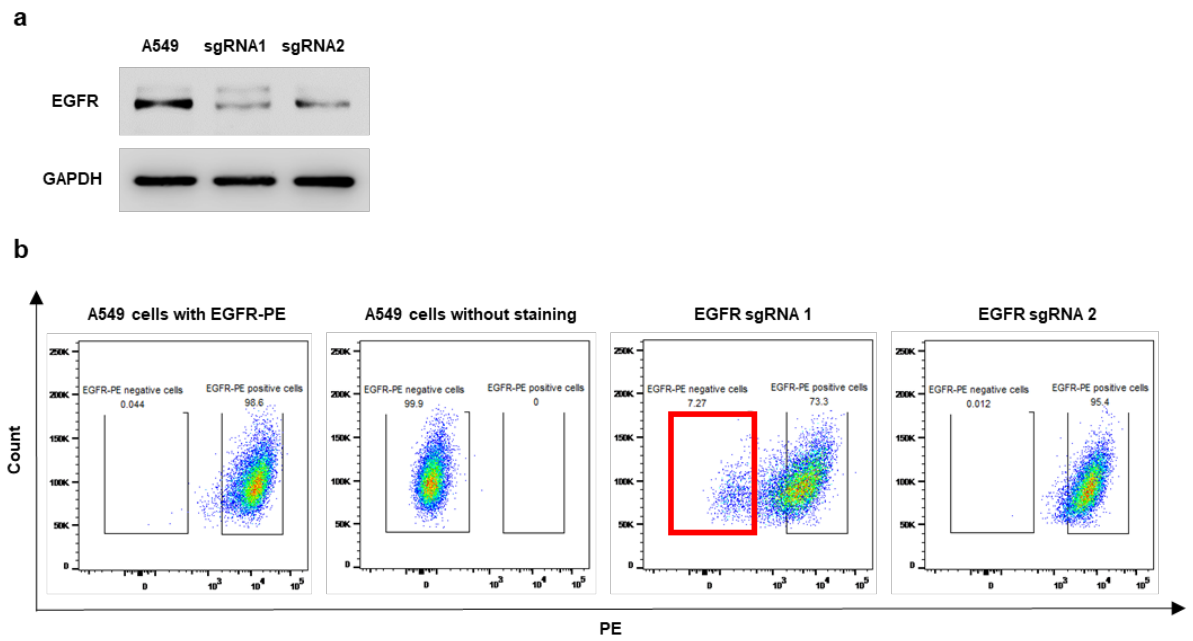

Supplementary Fig. 1. Construction of EGFR knockout cell line

a Transfection of A549 cells with epidermal growth factor receptor (*EGFR*) single guide (sg)RNA1 and sgRNA2. Western blotting was performed to detect the total EGFR and glyceraldehyde-3-phosphate dehydrogenase (GAPDH) (loading control) expression. The EGFR knockout status was confirmed by comparing EGFR expression levels.

b Staining of A549, A549 *EGFR* sgRNA1, and A549 *EGFR* sgRNA2 cells with or without EGFR-phycoerythrin (PE) antibody. Flow cytometry was used to identify EGFR knockout cells (red box).

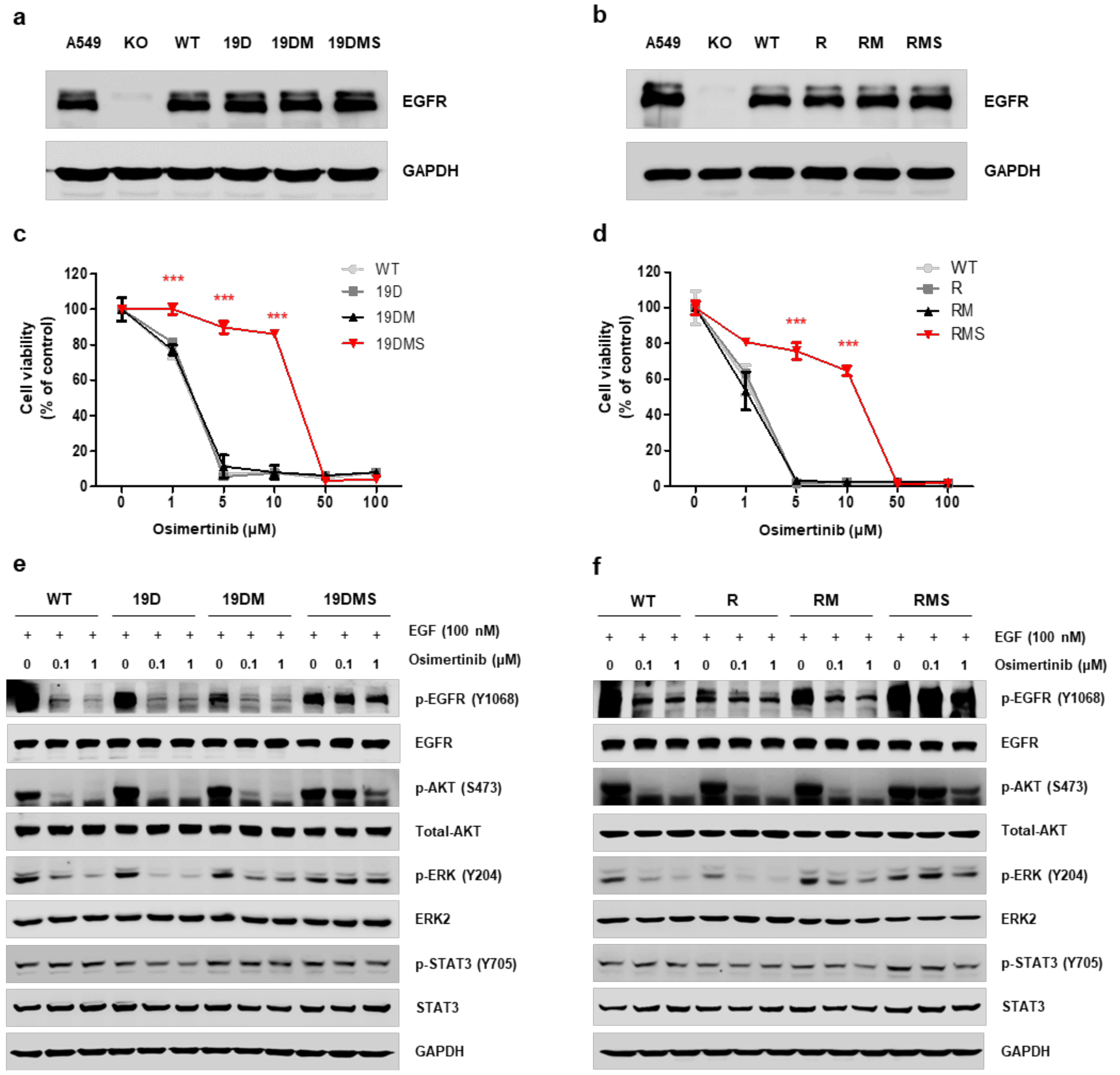

Supplementary Fig. 2 Addition of *EGFR* C797S mutation to A549 cells confers resistance to osimertinib, a third-generation EGFR-tyrosine kinase inhibitors (TKI)

EGFR^{WT} (WT), *EGFR*^{L858R} (R), *EGFR*$^{L858R+T790M}$ (RM), *EGFR*$^{L858R+T790M+C797S}$ (RMS), *EGFR*$^{19del747\_750}$ (19D), *EGFR*$^{19del747\_750+T790M}$ (19DM), or *EGFR*$^{19del747\_750+T790M+C797S}$ (19DMS) constructs were introduced into A549 *EGFR*^{KO} cells.

a, b Detection of epidermal growth factor receptor (EGFR) and glyceraldehyde 3-phosphate dehydrogenase (GAPDH) (loading control) expression using western blotting.

c, d Cells were treated with osimertinib at the indicated concentrations for 72 h. The cytotoxic

effect of osimertinib was assessed using the 3-(4,5-dimethylthiazol-2-yl)-2,5-diphenyl-2H-tetrazolium bromide (MTT) assay.

e, f Indicated cells were pre-treated with osimertinib (0, 1, and 10 µM) for 6 h, followed by epidermal growth factor (EGF) stimulation (100 ng/mL) for 5 min. Immunoblotting was performed to detect the levels of p-EGFR (Y1068), p- AKT Serine/Threonine Kinase 1 (AKT) (S473), p- extracellular signal-regulated kinase (ERK) (Y204), and p- signal transducer and activator of transcription 3 (STAT3) (Y705) as well as the expression of EGFR, total-AKT, ERK2, STAT3, and GAPDH (loading control).

Data information: Data presented in (**c** and **d**) represent the mean ± standard error of mean (SEM) and statistical analysis for conducted using Student's *t*-test, comparing with the control group (**c**, 19D; **d**, R). ***$p < 0.001$.

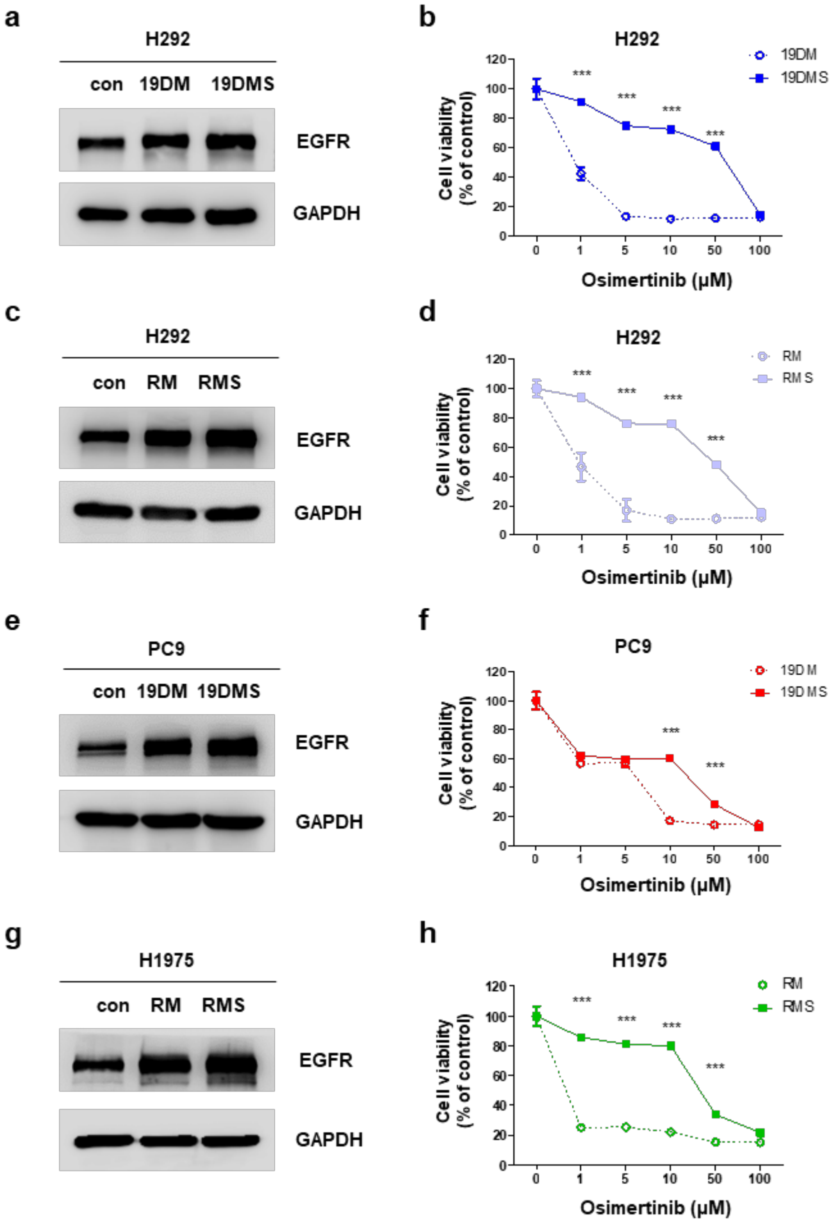

Supplementary Fig. 3 Amplification of *EGFR* C797S mutation in H292, H1975, and PC9 cells acquires resistance to osimertinib

H292 and PC9 cells consistently overexpressed epidermal growth factor receptor *(EGFR)* mutants ($EGFR^{19del747\_750+T790M}$ (19DM) or $EGFR^{19del747\_750+T790M+C797S}$ (19DMS)), whereas H292 and H1975 cells stably overexpressed *EGFR* mutants ($EGFR^{L858R+T790M}$ (RM)and $EGFR^{L858R+T790M+C797S}$ (RMS)).

a, c, e, g. Detection of EGFR and glyceraldehyde 3-phosphate dehydrogenase (GAPDH; loading control) expression using western blotting.

b, d, f, h. Indicated cells were treated with osimertinib for 72 h. The cytotoxic effect of osimertinib was assessed using the 3-(4,5-dimethylthiazol-2-yl)-2,5-diphenyl-2H-tetrazolium bromide (MTT) assay.

Data information: Data presented in (**b**, **d**, **f**, and **h**) represent the mean ± standard error of mean (SEM) and statistical analysis for was conducted using Student's *t*-test, comparing with the control group (**b**, 19DM; **d**, RM; **f**, 19DM; **h**, RM). ***p < 0.001.

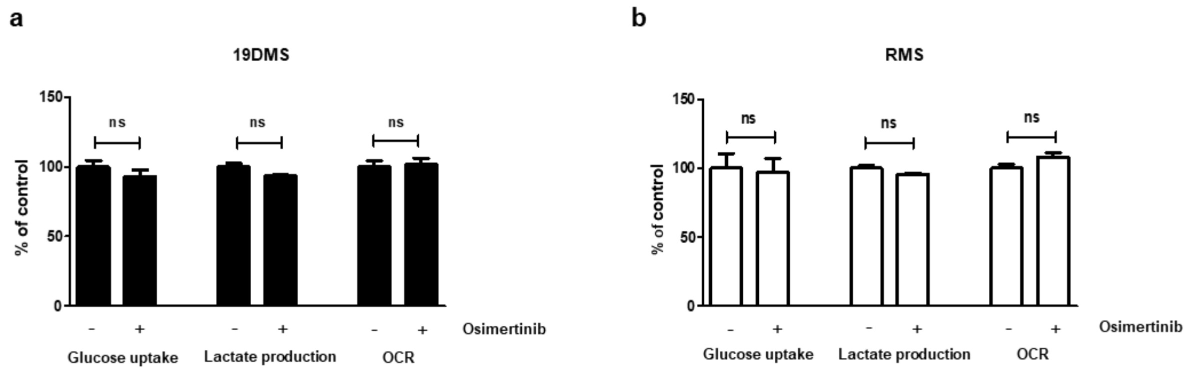

Supplementary Fig. 4 Osimertinib has no effect on glycolysis in A549 cells with the epidermal growth factor receptor (*EGFR*) C797S mutation

A549 *EGFR*$^{19del747\_750+T790M+C797S}$ (19DMS) cells (**a**) and A549 *EGFR*$^{L858R+T790M+C797S}$ (RMS) cells (**b**) were treated with osimertinib (1 μM) for 6 h. Glucose uptake, lactate production, and oxygen consumption rate (OCR) were measured.

Data information: The data presented in (**a** and **b**) represent the mean ± standard error of mean (SEM). Statistical analysis (**a** and **b**) was conducted using Student's *t*-test and compared with control groups. "ns" indicates no statistical significance.

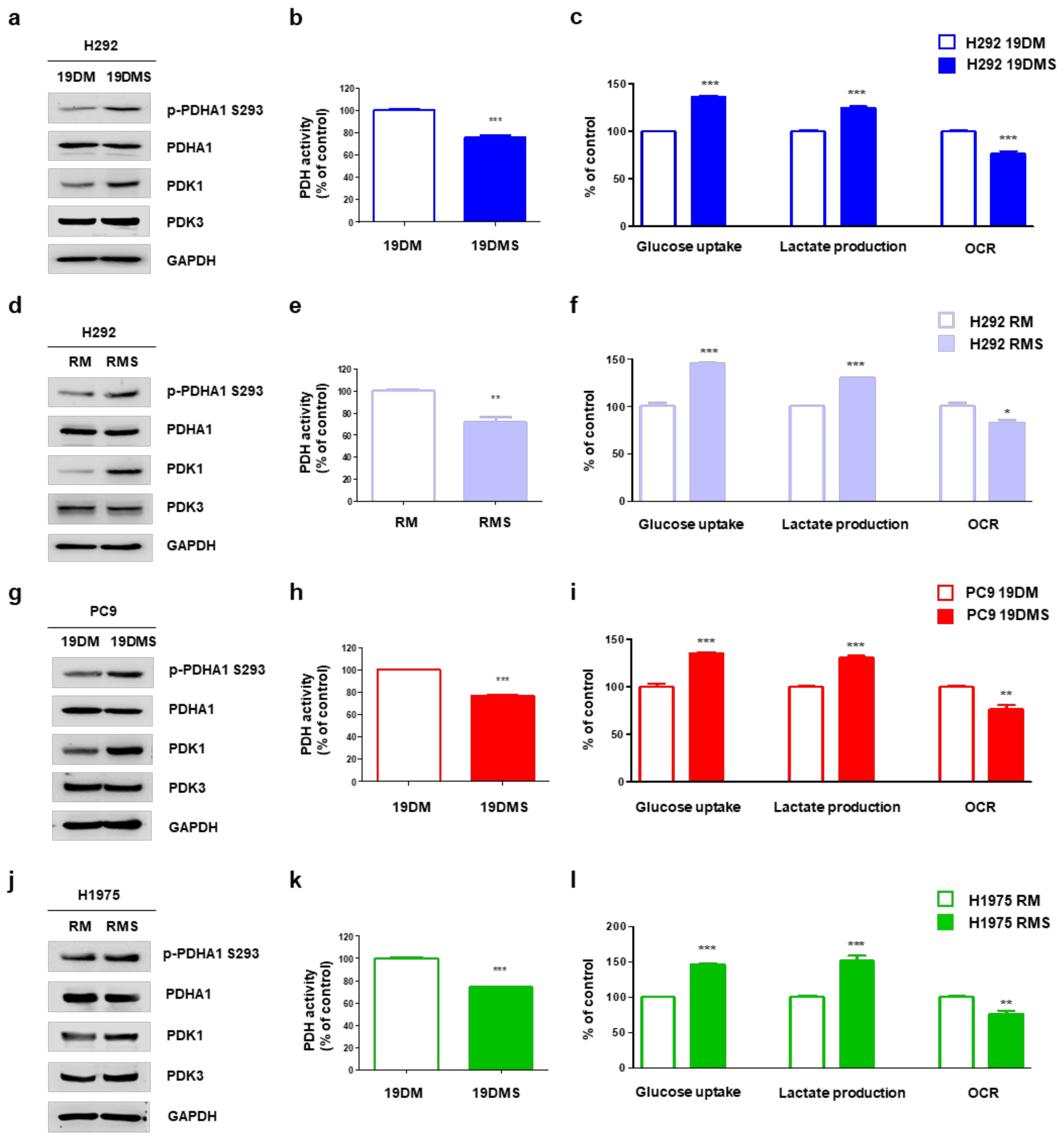

Supplementary Fig. 5 Epidermal growth factor receptor *(EGFR)* C797S mutation induces PDK1 upregulation and promotes glycolysis in H292, H1975, and PC9 cells

a, **d, g, j** p-pyruvate dehydrogenase E1 subunit alpha 1(PDHA1) (S293), total PDHA1, pyruvate dehydrogenase kinase (PDK)1, PDK3, and glyceraldehyde 3-phosphate dehydrogenase (GAPDH, loading control) expression in the indicated cells was detected using western blotting.

b, e, h, k Pyruvate dehydrogenase (PDH) activity was measured in indicated cells.

c, f, i, l Glucose uptake, lactate production, and oxygen consumption rate (OCR) were measured in the indicated cells.

Data information: The data presented in (**b**, **c**, **e**, **f**, **h**, **i**, **k**, and **l**) represent the mean ± standard error of mean (SEM). Statistical analysis for (**b**, **c**, **e**, **f**, **h**, **i, k**, and **l**) was conducted using Student's *t*-test and compared with the control group (**b** and **c**, 19DM; **e** and **f**, RM; **h** and **i**, 19DM; **k** and **l**, RM). *$p < 0.05$, **$p < 0.01$, and ***$p < 0.001$.

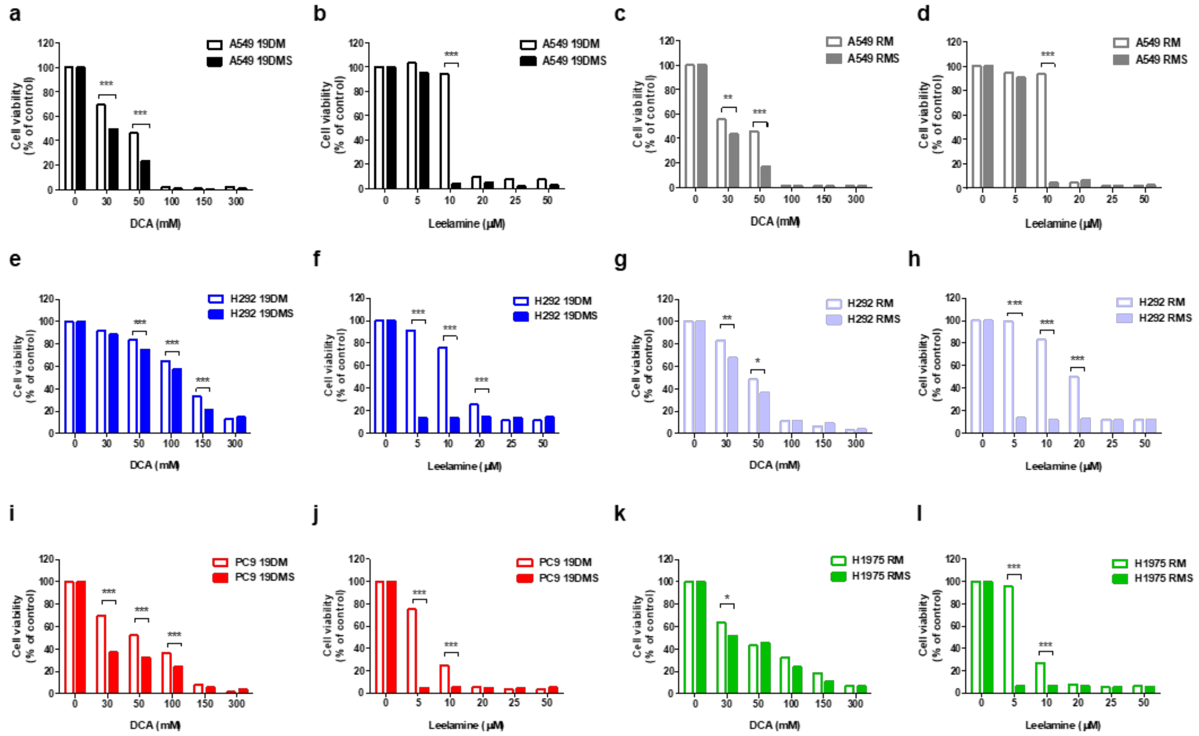

Supplementary Fig. 6 A549 *EGFR* C797S mutant cells are sensitive to PDK inhibitors

A549 epidermal growth factor receptor *(EGFR)* mutant cells were treated with dichloroacetate (DCA) or leelamine at the indicated concentrations for 72 (A and C) or 24 h (B and D–L).

a–l The cytotoxic effects of the indicated PDK inhibitors were assessed using the 3-(4,5-dimethylthiazol-2-yl)-2,5-diphenyl-2H-tetrazolium bromide (MTT) assay.

Data information: Data presented in (**a–l**) represent the mean ± standard error of mean (SEM) and statistical analysis for was conducted using Student's *t*-test, comparing with the 19DM or RM group. *$p < 0.05$, **$p < 0.01$, and ***$p < 0.001$.

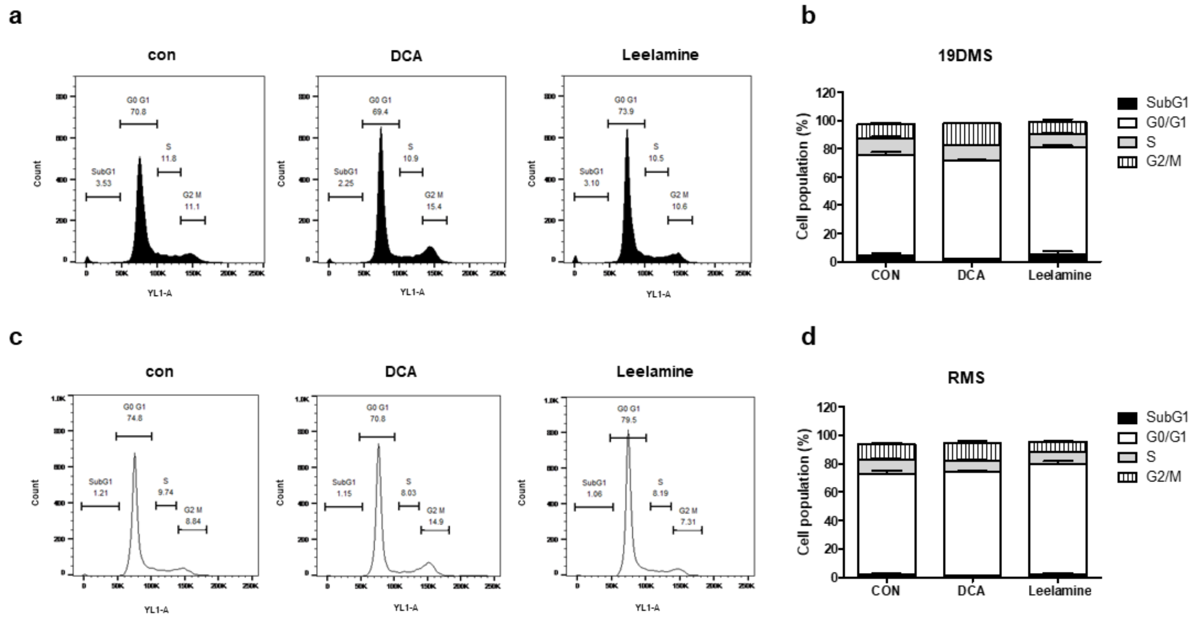

Supplementary Fig. 7 DCA and leelamine do not cause cell cycle arrest or affect cell proliferation in A549 cells with epidermal growth factor receptor *(EGFR)* C797S mutation

A549 $EGFR^{19del747\_750+T790M+C797S}$ (19DMS) cells (**a** and **b**) and A549 $EGFR^{L858R+T790M+C797S}$ (RMS) cells (**c** and **d**) were treated with DCA (50 mM) or leelamine (10 µM) for 15 h.

a, c Representative histograms showing cell cycle distribution obtained by flow cytometry.

b, d Analysis of cell population at each cell cycle phase compared to the total phase.

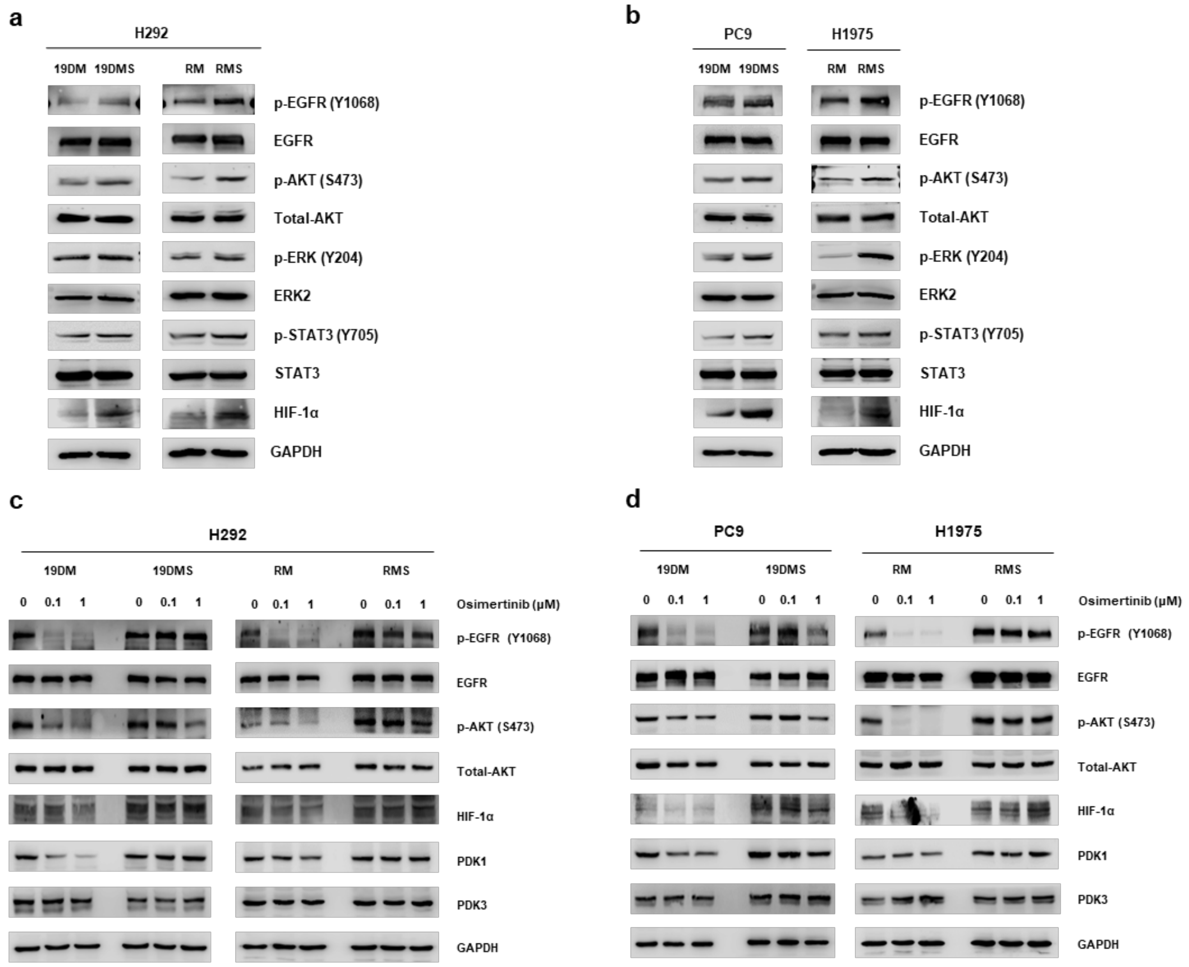

Supplementary Fig. 8 *EGFR* C797S mutation activates the EGFR/AKT/HIF-1α axis, leading to increased PDK1 expression in H292, PC9, and H1975 cells, with limited regulation by osimertinib

a, b Immunoblotting analysis was performed to detect the levels of p-epidermal growth factor receptor (EGFR) (Y1068), p-AKT Serine/Threonine Kinase 1 (AKT) (S473), p-extracellular signal-regulated kinase (ERK) (Y204), and p-signal transducer and activator of transcription 3 (STAT3) (Y705), as well as the expression of EGFR, total-AKT, ERK2, STAT3, hypoxia-inducible factor (HIF)-1α, and glyceraldehyde 3-phosphate dehydrogenase (GAPDH, loading control).

c, d The indicated cells were treated with osimertinib (0, 0.1, and 1 μM) for 24 h.

Immunoblotting analysis was performed to assess the levels of p-EGFR (Y1068) and p-AKT (S473), as well as the expression of EGFR, total-AKT, HIF-1α, PDK1, PDK3, and GAPDH (loading control).

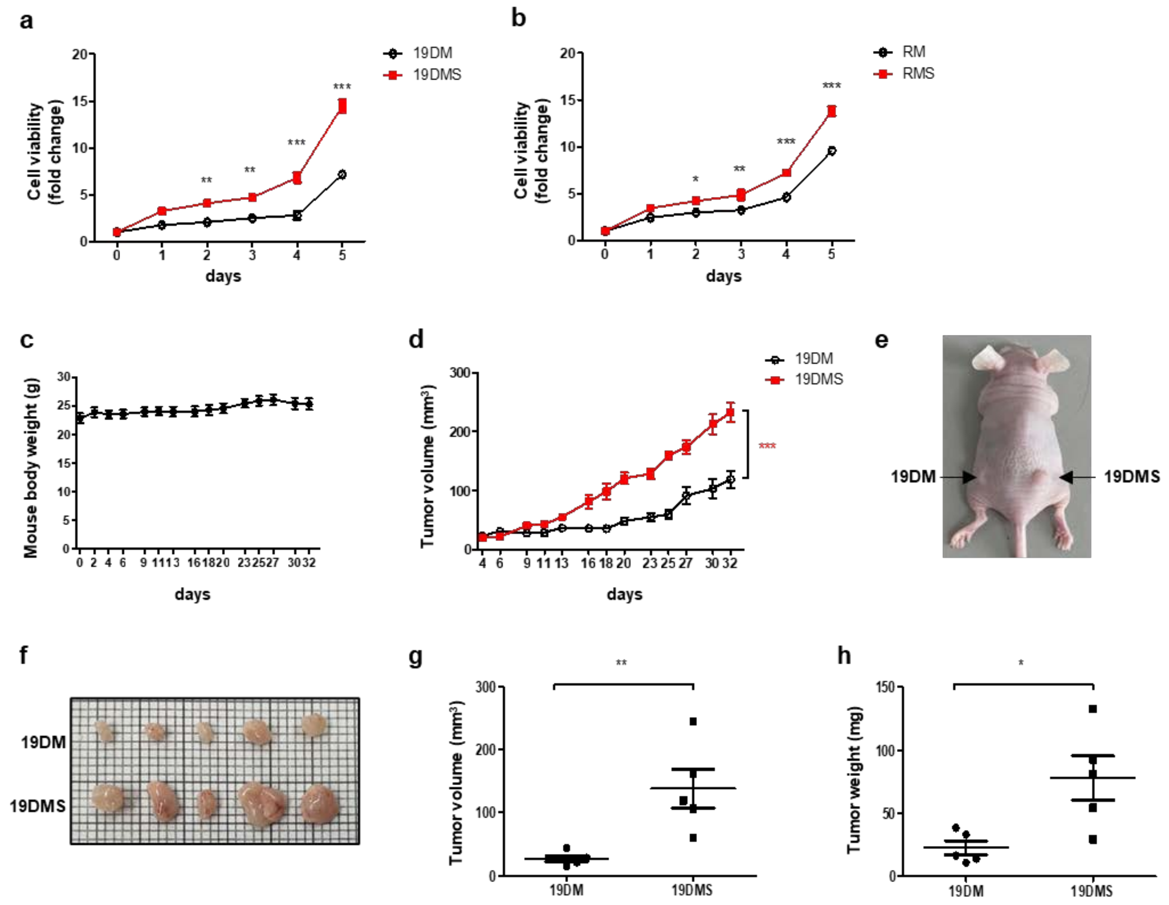

Supplementary Fig. 9 Epidermal growth factor receptor *(EGFR)* C797S mutation amplifies cell proliferation in A549 cells

a, b Cell proliferation was assessed using 3-(4,5-dimethylthiazol-2-yl)-2,5-diphenyl-2H-tetrazolium bromide (MTT)assay (**a**, A549 $EGFR^{L858R+T790M}$ (RM)/ $EGFR^{L858R+T790M+C797S}$ (RMS) cells; **b**, A549 $EGFR^{19del747\_750+T790M}$ (19DM)/$EGFR^{19del747\_750+T790M+C797S}$ (19DMS) cells).

c–h A549 19DM (left) and A549 19DMS (right) cells were injected into BALB/c nude mice. Body weight (**c**) and tumor volume (**d**) were measured during the treatment period. Images of the mice (**e**) and tumors (**f**) were acquired. Tumor volume (**g**) and weight (**h**) were also analyzed. Data information: Data presented as (**a**, **b**, **d**, **g**, and **h**) represent the mean ± standard error of mean (SEM). Statistical analyses for (**a** and **b**) were conducted using Student's *t*-test and compared with the control group (**a**, 19DM; **b**, RM). Statistical analyses for (**d**, **g**, and **h**) were

performed using Student's *t*-test and compared with the 19DM group. *$p < 0.05$, **$p < 0.01$, and ***$p < 0.001$.

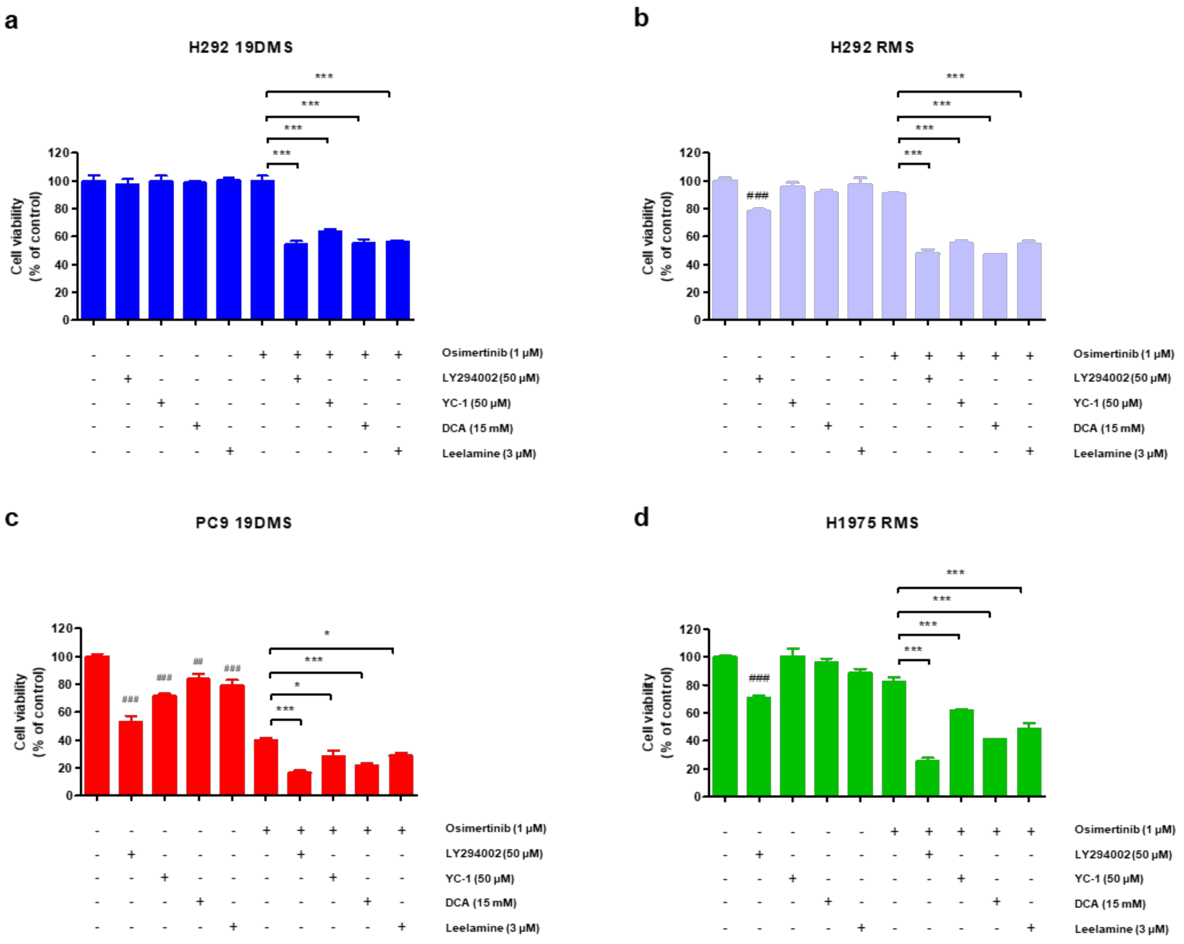

Supplementary Fig. 10 Combining inhibitors targeting PI3K, HIF-1α, or PDK1 alongside osimertinib enhances its efficacy in H292, H1975, and PC9 cells with the *EGFR* C797S mutation

a–d. Indicated cells treated with osimertinib (1 μM) in combination with or without LY294002 (50 μM), YC-1 (50 μM), DCA (15 mM), or leelamine (3 μM) for 24 h. Cell viability was assessed using the 3-(4,5-dimethylthiazol-2-yl)-2,5-diphenyl-2H-tetrazolium bromide (MTT) assay.

Data information: The data presented in (**a–d**) represent the mean ± standard error of mean (SEM). Statistical analysis for (**a–d**) was conducted using one-way analysis of variance with Dunnett's post-hoc test and compared with the osimertinib treatment group (the sixth lane). **p* < 0.05 and ****p* < 0.001. Statistical analysis for (**b–d**) was performed using one-way analysis

of variance with Dunnett's post-hoc test, compared to the control group (the first lane of 19DMS or RMS, respectively). ##$p < 0.01$ and ###$p < 0.001$.

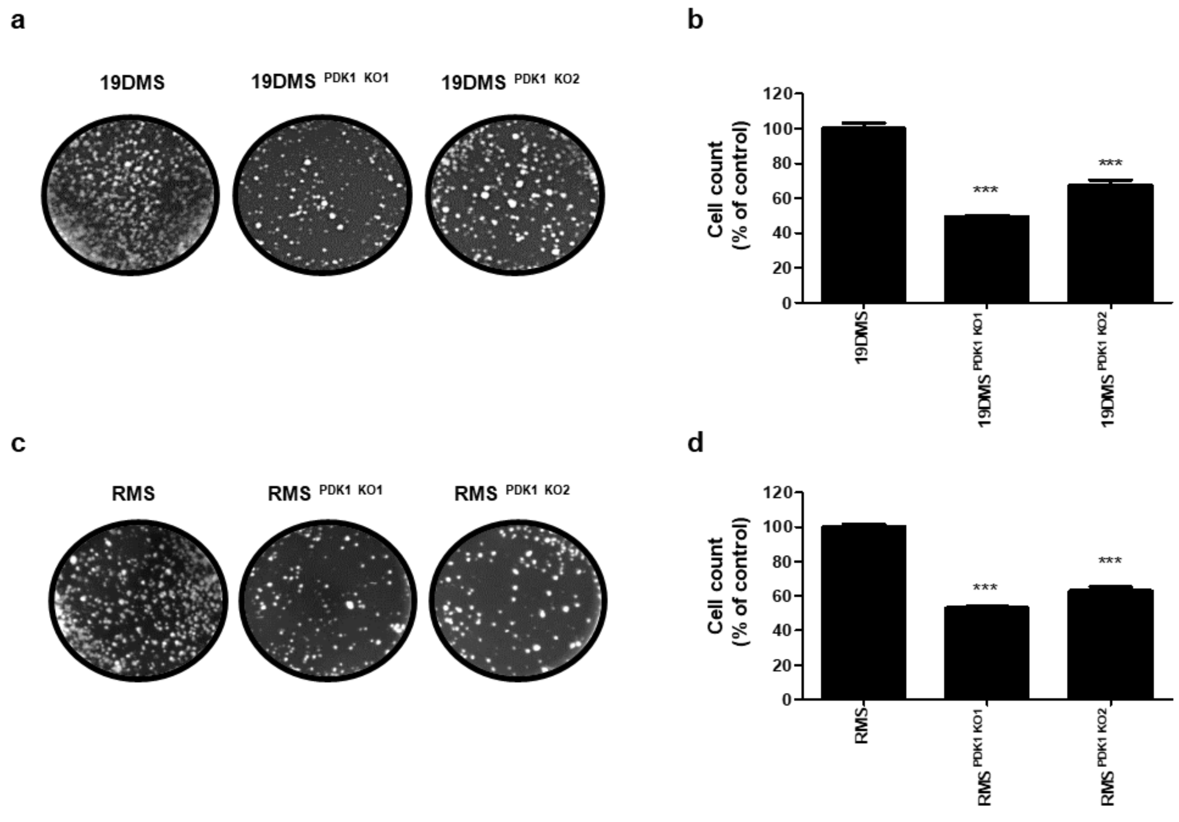

Supplementary Fig. 11 Pyruvate dehydrogenase kinase 1 (PDK1) knockout successfully inhibits cell growth in A549 cells harboring the *EGFR* C797S mutation

a, b Colony formation assays were performed using A549 $EGFR^{19del747\_750+T790M+C797S}$ (19DMS), A549 $19DMS^{PDK1\ KO1}$, and A549 $19DMS^{PDK1\ KO2}$ cells.

c, d. Colony formation assays performed using A549 RMS, A549 $RMS^{PDK1\ KO1}$, and A549 $RMS^{PDK1\ KO2}$ cells.

KO; knockout.

Data information: Cells were cultured for 2 weeks, and the results (**b** and **d**) are presented as the mean ± standard error of mean (SEM) to evaluate cell proliferation. Statistical analysis was performed using a one-way analysis of variance with Dunnett’s test. ***$p < 0.001$, compared with the control group (**b**, 19DMS; **d**, RMS).

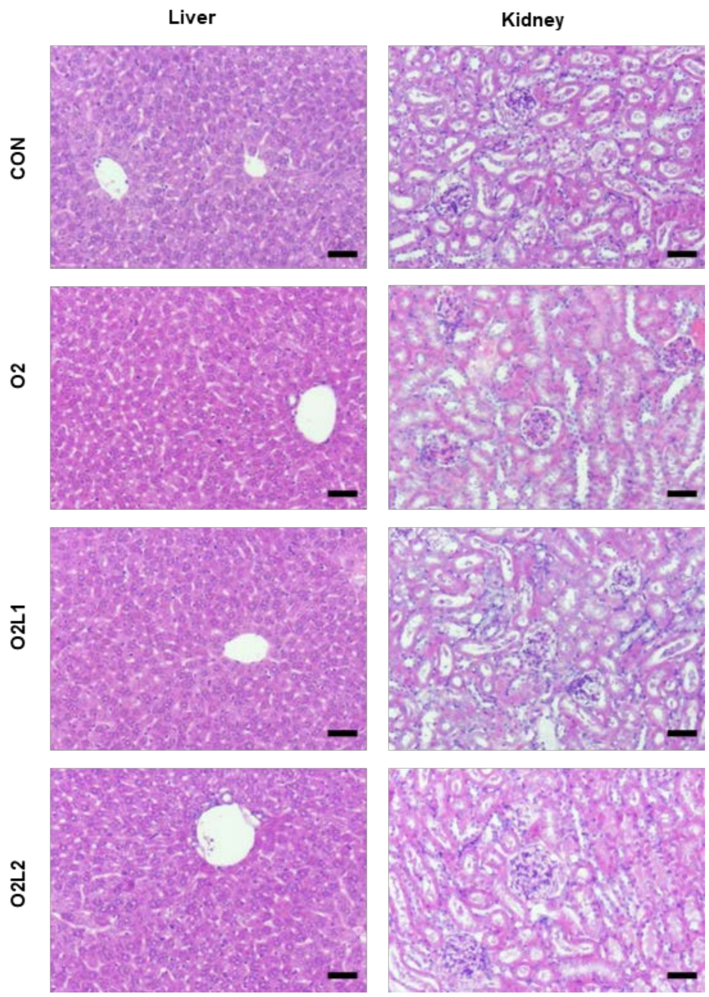

Supplementary Fig. 12 Osimertinib and leelamine do not induce renal or hepatic cytotoxicity in allograft models

The allograft models were established in C57BL/6 mice by injecting a 1:1 mixture of LLC 19DM-BFP and LLC 19DMS-GFP cells. After one week, the mice were divided into the following treatment groups: corn oil (CON), osimertinib 2 mg/kg (O2), osimertinib 2 mg/kg + leelamine 1 mg/kg (O2L1), or osimertinib 2 mg/kg + leelamine 2 mg/kg (O2L2). These treatments were administered for two weeks. Subsequently, the kidney and liver samples were collected and stained with hematoxylin and eosin (HE) to evaluate the cytotoxic effects of the administered drugs. Magnification was set to 100×, and the scale bar represents 200 μm.

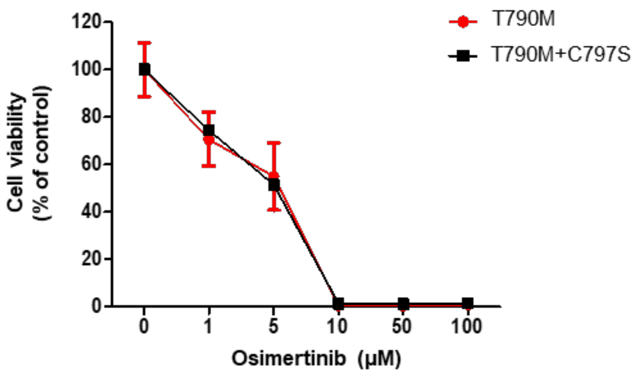

Supplementary Fig. 13 Epidermal growth factor receptor *(EGFR)* T790M + C797S mutation do not induce osimertinib resistance in A549 cells

EGFR T790M or *EGFR* T790M+C797S constructs were introduced into A549 $EGFR^{KO}$ cells. Cells were treated with osimertinib at indicated concentrations for 72 h. The cytotoxic effect of osimertinib was assessed using the 3-(4,5-dimethylthiazol-2-yl)-2,5-diphenyl-2H-tetrazolium bromide (MTT) assay.

KO; knockout.

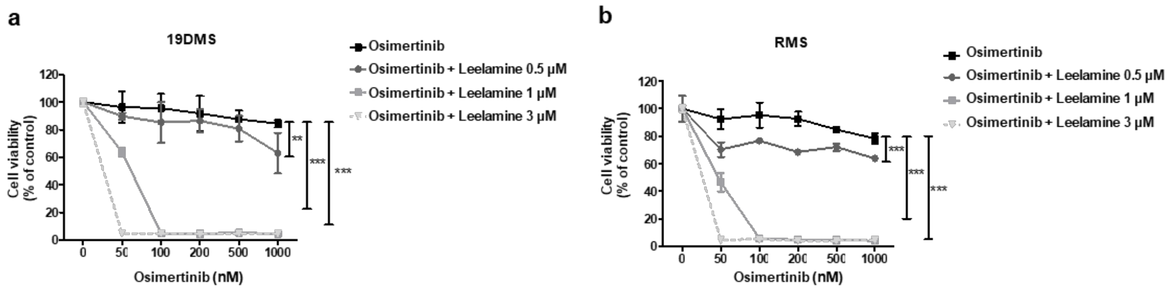

Supplementary Fig. 14 Leelamine significantly enhances the sensitivity of A549 cells with epidermal growth factor receptor *(EGFR)* C797S mutation to osimertinib

A549 $EGFR^{19del747\_750+T790M+C797S}$ (19DMS) (**a**) and $EGFR^{L858R+T790M+C797S}$ (RMS) (**b**) cells were treated with osimertinib with or without leelamine as indicated for 48 h. The cell viability was assessed using the 3-(4,5-dimethylthiazol-2-yl)-2,5-diphenyl-2H-tetrazolium bromide (MTT) assay.

Statistical analysis for (**a** and **b**) was conducted using Student's *t*-test and compared with the control (osimertinib) group. $^{**}p < 0.01$ and $^{***}p < 0.001$.

Supplementary Tables

Supplementary Table 1. List of antibodies

Antibodies	Manufacturer	Catalog number	Dilution ratio
Anti-mouse IgG	Invitrogen	#RJ240410	1:4000
Anti-rabbit IgG	Invitrogen	#SA245916	1:4000
Beta (β)-Actin	Sigma	#A2066	1:1000
p-AKT (S473)	Abcam	ab81283	1:1000
AKT 1+2+3 (total-AKT)	Abcam	ab32505	1:1000
p-EGFR (Y1068)	Abcam	ab40815	1:1000
EGFR	Abcam	ab52894	1:1000
EGFR-PE	Abcam	ab27764	1:1000
p-ERK (Y204)	Santa Cruz	sc-7383	1:1000
ERK2	Santa Cruz	sc-154	1:1000
GAPDH	Santa Cruz	sc-47724	1:1000
HIF-1α	Novus	NB100-105	1:1000
HSP90	Santa Cruz	sc-69703	1:1000
p-PDHA1(S232)	MERCK	AP1063	1:1000
p-PDHA1(S293)	Abcam	ab177461	1:1000
p-PDHA1(S300)	MERCK	AP1064	1:1000
PDHA1	Santa Cruz	sc-377092	1:1000
PDK1	Enzo	ADI-KAP-PK112	1:1000
PDK2	Proteintech	15647-1-AP	1:1000
PDK3	Abcam	ab154549	1:1000
PDK4	Proteintech	12949-1-AP	1:1000

p-STAT3 (Y705)	Cell Signaling Technology	#9138	1:1000
STAT3	Santa Cruz	sc-482	1:1000

Supplementary Table 2. List of reagents

Reagents	Manufacturer	Catalog number
2-(7-Nitro-2,1,3-benzoxadiazol-4-yl)-D-glucosamine (2-NBDG)	Thermo Fisher Scientific Inc.	N13195
3-(4,5-Dimethylthiazol-2-yl)2,5-diphenyltetrazolium bromide (MTT)	Abcam	ab7764
Blasticidin	Sigma-Aldrich	15205
DCA	Sigma-Aldrich	347795
EGF	Santa Cruz	sc-47724
HCS NuclearMask Stains	Thermo Fisher Scientific Inc.	H10326
Hygromycin B	Sigma-Aldrich	10843555001
Leelamine	Abcam	ab120923
Lipofectamine2000	Invitrogen	11668019
LY294002	Abcam	ab40815
Osimertinib	Abcam	ab52894
Penicillin/streptomycin	Gibco	15140122
Polybrene	Sigma-Aldrich	TR-1003
Polyetherimide	Sigma-Aldrich	700193
Puromycin	Sigma-Aldrich	P9620
Tofacitinib	Abcam	ab32505
U0126	Abcam	ab81283

Supplementary Table 3. List of single guide RNAs (sgRNAs)

sgRNAs	Targeting DNA sequences
EGFR #1	5´-TCCTCCAGAGCCCGACTCGC-3´ (exon 1)
EGFR #2	5´-AGTAACAAGCTCACGCAGTT-3´ (exon 2)
PDK1 #1	5´-GAACTGCTTCATGGAGAGCG-3´ (exon 1)
PDK1 #2	5´-GTCATTCCCACAATGGCCCA-3´ (exon 4)

EGFR: epidermal growth factor receptor; PDK1: pyruvate dehydrogenase kinase 1.

Supplementary Table 4. List of primers

Primers	Sequences	Purpose
EGFR 19del_747-750 – F	5´-CGTCGCTATCAAGGAAACATCTCCGAAAGCCAACAAGGAAATCCTC-3´	Deletion mutagenesis
EGFR 19del_747-750 – R	5´-GAGGATTTCCTTGTTGGCTTTCGGAGATGTTTCCTTGATAGCGACG-3´	Deletion mutagenesis
EGFR C797S – F	5´-CATGCCCTTCGGCTCCCTCCTGGAGCTA-3´	Single amino acid mutagenesis
EGFR C797S – R	5´-TAGCTCCAGGAGGGAGCCGAAGGGCATG-3´	Single amino acid mutagenesis
EGFR CDS – F	5´-AATGCGTGGACAAGTGCAAC-3´	qRT-PCR
EGFR CDS – R	5´-GCTTCGGGCCATTTTGGAGA-3´	qRT-PCR

EGFR: epidermal growth factor receptor; F: forward; R: reverse; qRT-PCR: real-time quantitative reverse transcription PCR

Supplementary Table 5. Patient characteristics and treatment details

Patient No.	Pathology No.	Group	Sex (M/F)	Age (Years)	Surgical approach	Pathologic staging	Duration of osimertinib administration (Months)	Enrolled reason	Gene mutation
P1	827487-7D	Osimertinib-sensitive	F	56	RUL lobectomy	pT2aN0M0, IB	29	No severe drug-related adverse effects; No recurrence; No metastasis	*EGFR* p.L858R (+), TP53 (+
P2	856835-4B	Osimertinib-sensitive	F	65	RML lobectomy	pT2aN2M0, IIIA	24	No severe drug-related adverse effects; No recurrence;	*EGFR* p.L858R (+), TP53 (+)

							No metastasis	
P3	858082-7A	Osimertinib-sensitive	F	51	LUL lobectomy	pT2aN1M0, IIB	23	No severe drug-related adverse effects; No recurrence; No metastasis	*EGFR* p.L858R (+)
P4	863049-10C	Osimertinib-sensitive	F	65	RLL lobectomy	pT2aN1M0, IIB	23	No severe drug-related adverse effects; No recurrence; No metastasis	*EGFR* p.L858R (+)

P5	864107-9B	Osimertinib-sensitive	M	61	LUL lobectomy + WR of LLL	pT2aN1M0, IIB	19	No severe drug-related adverse effects; No recurrence; No metastasis	*EGFR* p.E746_P753delinsLS (+)
P6	866283-10A	Osimertinib-sensitive	M	60	RUL lobectomy + WR of RLL	pT1cN2M0, IIIA	22	No severe drug-related adverse effects; No recurrence; No metastasis	*EGFR* p.L858R (+), TP53 (+)
P7	868253-12B	Osimertinib-sensitive	F	55	LUL lobectomy	pT2aN2M0, IIIA	17	No severe drug-related adverse	*EGFR* p.E746-A750del (+); EGFR p.E746-S752delinsV (+), TP53 (+)

					+ WR of RLL			effects; No recurrence; No metastasis	
P8	882335-5D	Osimertinib-sensitive	F	67	RLL lobectomy	pT1cN0M0, IA3	16	No severe drug-related adverse effects; No recurrence; No metastasis	*EGFR* 19-Del (+)
P9	892107-7A	Osimertinib-sensitive	F	35	RML lobectomy	pT1bN0M1a, IVA	16	No severe drug-related adverse effects; No recurrence;	*EGFR* 19-Del (+)，*EGFR/ERBB2/MET/CDK4* amplification, TP53 (+)

								No metastasis	
P10	897298-6C	Osimertinib-sensitive	M	47	RUL lobectomy + WR of RML	pT3N0M0, IIB	20	No severe drug-related adverse effects; No recurrence; No metastasis	*EGFR* p.L858R (+)
P11	839430-8C	Osimertinib-resistant	M	48	LLL lobectomy	pT2aN0M0, IB	3	bone metastasis	*EGFR* p.L858R (+), TP53 (+)
P12	853853-S1A	Osimertinib-resistant	F	33	WR of LLL	pT4NxM1a, IVA	2	Severe rash and pruritus: Bone metastasis	*EGFR* p.L858R (+)
P13	855501-8D	Osimertinib-resistant	M	61	RUL lobectomy	pT1bN0M0, IA2	5	Lymph nodes metastasis	*EGFR* p.L858R (+)

P14	856564-8B	Osimertinib-resistant	F	64	LUL lobectomy	pT2aN0M0, IB	8	Pulmonary metastases	*EGFR* 19-Del (+)
P15	859304-10B	Osimertinib-resistant	M	67	RUL lobectomy + WR of LUL	pT2aN0M0, IB	2	Bone metastasis	*EGFR* p.L858R (+); *EGFR* T790M (+)
P16	859684-3A	Osimertinib-resistant	M	40	WR of RLL + Chest wall nodules resection	pT4NxM1a IVA	19	Pulmonary metastases	*EGFR* p.L858R (+)
P17	870807-E	Osimertinib-resistant	F	59	WR of RLL + Chest wall nodules resection	pT1cNxM1a IVA	4	Bone metastasis	*EGFR* 20-Ins (+)

P18	880321-10A	Osimertinib-resistant	F	52	RUL lobectomy	pT1cN3M0, IIIB	3	Bone metastasis	*EGFR* 19-Del (+)
P19	883007-7C	Osimertinib-resistant	F	55	RUL lobectomy	pT1bN0M0, IA2	4	Bone metastasis	*EGFR* 19-Del (+)
P20	883395-SL	Osimertinib-resistant	F	56	LUL lobectomy	pT1N2M0, IIIA	11	Severe diarrhea; Liver dysfunction	*EGFR* p.L858R (+)

Supplementary Table 6. Immunohistochemical analysis of p-PDHA and PDK1 expression through in tissue samples of patients with non-small cell lung cancer.

Patient No.	Pathology No.	Group	p-PDHA (S293) (positive rate/staining intensity)	PDK1 (positive rate/staining intensity)
P1	827487-7D	Osimertinib-sensitive	0.6/2	0.08/1
P2	856835-4B	Osimertinib-sensitive	0.75/2	0.7/2
P3	858082-7A	Osimertinib-sensitive	0.7/2	0.5/2
P4	863049-10C	Osimertinib-sensitive	0.5/3	0.2/2
P5	864107-9B	Osimertinib-sensitive	0.75/3	0.4/2
P6	866283-10A	Osimertinib-sensitive	0.7/2	0.7/2
P7	868253-12B	Osimertinib-sensitive	0.6/2	0.8/2
P8	882335-5D	Osimertinib-sensitive	0.85/3	0.6/3
P9	892107-7A	Osimertinib-sensitive	0.5/2	0.8/2
P10	897298-6C	Osimertinib-sensitive	0.2/1	0/0
P11	839430-8C	Osimertinib-resistant	0.8/3	0.6/2

P12	853853-S1A	Osimertinib-resistant	1/3	0.95/2
P13	855501-8D	Osimertinib-resistant	1/3	0.9/2
P14	856564-8B	Osimertinib-resistant	1/3	0.9/2
P15	859304-10B	Osimertinib-resistant	0.95/3	0.9/3
P16	859684-3A	Osimertinib-resistant	1/3	0.8/2
P17	870807-E	Osimertinib-resistant	0.7/3	0.85/3
P18	880321-10A	Osimertinib-resistant	0.9/3	0.7/2
P19	883007-7C	Osimertinib-resistant	0.85/3	0.7/2
P20	883395-SL	Osimertinib-resistant	1/3	0.7/2

p-PDHA**:** phosphorylated pyruvate dehydrogenase E1 subunit alpha 1; PDK1**:** pyruvate dehydrogenase kinase 1**.**

Supplementary Table 7. CC_{50} values of osimertinib in A549 *EGFR*-mutant cells

Cell lines	CC_{50} values (μM)
WT	1.49 (1.33–1.67)
19D	1.30 (0.37–4.50)
19DM	1.59 (1.41–1.78)
19DMS	16.61 (13.21–20.89)
R	1.10 (0.66–1.84)
RM	1.06 (0.95–1.12)
RMS	11.80 (8.86–15.72)

CC_{50}: 50% cytotoxicity concentration; EGFR: epidermal growth factor receptor; Mutations: *$EGFR^{WT}$* (WT); *$EGFR^{L858R}$* (R); *$EGFR^{L858R+T790M}$* (RM); *$EGFR^{L858R+T790M+C797S}$* (RMS); *$EGFR^{19del747\_750}$* (19D); *$EGFR^{19del747\_750+T790M}$* (19DM); *$EGFR^{19del747\_750+T790M+C797S}$* (19DMS). The values in parentheses represent the 95% confidence intervals associated with the CC_{50} values.

Supplementary Table 8. CC_{50} values of osimertinib in H292, PC9, and H1975 *EGFR*-mutant cells

Cell lines-mutations	CC_{50} values (μM)
H292-19DM	0.74 (0.64–0.86)
H292-19DMS	22.02 (11.68–41.51)
H292-RM	0.77 (0.60–0.99)
H292-RMS	19.26 (12.64–29.35)
PC9-19DM	1.40 (0.66–2.99)
PC9-19DMS	4.45 (2.25–8.81)
H1975-RM	0.18 (0.11–0.29)
H1975-RMS	15.87 (11.34–22.20)

CC_{50}: 50% cytotoxicity concentration; EGFR: epidermal growth factor receptor; Mutations: $EGFR^{L858R}$ (R); $EGFR^{L858R+T790M}$ (RM); $EGFR^{L858R+T790M+C797S}$ (RMS); $EGFR^{19del747\_750}$ (19D); $EGFR^{19del747\_750+T790M}$ (19DM); $EGFR^{19del747\_750+T790M+C797S}$ (19DMS). The values in parentheses represent the 95% confidence intervals associated with the CC_{50} values.

Supplementary Table 9. CC_{50} values of DCA in *EGFR*-mutant cells

Cell lines-mutations	CC_{50} values (mM)
A549-19DM	43.07 (39.68–46.4)
A549-19DMS	30.16 (28.37–32.06)
A549-RM	36.88 (32.06–42.43)
A549-RMS	27.30 (25.78–28.91)
H292-19DM	102.8 (93.95–112.5)
H292-19DMS	80.04 (69.41–92.29)
H292-RM	47.83 (45.72–50.05)
H292-RMS	38.96 (37.49–40.48)
PC9-19DM	52.44 (45.58–60.35)
PC9-19DMS	19.34 (13.31–28.11)
H1975-RM	40.05 (34.90–45.97)
H1975-RMS	32.69 (27.76–38.50)

CC_{50}: 50% cytotoxicity concentration; EGFR: epidermal growth factor receptor; DCA: dichloroacetate; Mutations: $EGFR^{L858R}$ (R); $EGFR^{L858R+T790M}$ (RM); $EGFR^{L858R+T790M+C797S}$ (RMS); $EGFR^{19del747\_750}$ (19D); $EGFR^{19del747\_750+T790M}$ (19DM); $EGFR^{19del747\_750+T790M+C797S}$ (19DMS). The values in parentheses represent the 95% confidence intervals associated with the CC_{50} values.

Supplementary Table 10. CC_{50} values of leelamine in *EGFR*-mutant cells

Cell lines-mutations	CC_{50} values (μM)
A549-19DM	13.01 (10.82–15.65)
A549-19DMS	6.78 (6.38–7.20)
A549-RM	13.23 (12.01–14.57)
A549-RMS	6.49 (6.03–6.99)
H292-19DM	12.69 (11.94–13.49)
H292-19DMS	0.02 (0.01–0.16)
H292-RM	16.13 (14.12–18.42)
H292-RMS	0.08 (0.01–4.98)
PC9-19DM	6.83 (6.63–7.04)
PC9-19DMS	0.01 (0.01–0.07)
H1975-RM	8.15 (7.86–8.45)
H1975-RMS	0.06 (0.02–0.14)

CC_{50}: 50% cytotoxicity concentration; EGFR: epidermal growth factor receptor; Mutations: $EGFR^{WT}$ (WT); $EGFR^{L858R}$ (R); $EGFR^{L858R+T790M}$ (RM); $EGFR^{L858R+T790M+C797S}$ (RMS); $EGFR^{19del747\_750}$ (19D); $EGFR^{19del747\_750+T790M}$ (19DM); $EGFR^{19del747\_750+T790M+C797S}$ (19DMS). The values in parentheses represent the 95% confidence intervals associated with the CC_{50} values.

Supplementary Table 11. CC_{50} values of osimertinib in A549 EGFR *PDK1* knockout cells mutant cells

Cell lines	CC_{50} values (μM)
19DMS	16.63 (13.22–20.90)
$19DMS^{PDK1\ KO1}$	2.99 (1.78–5.03)
$19DMS^{PDK1\ KO2}$	2.97 (1.56–5.68)
RMS cells	14.34 (10.82–19.00)
$RMS^{PDK1\ KO1}$	1.00 (0.88–1.14)
$RMS^{PDK1\ KO2}$	1.09 (0.93–1.29)

CC_{50}: 50% cytotoxicity concentration; EGFR: epidermal growth factor receptor; PDK1: pyruvate dehydrogenase kinase 1; KO: knockout. Mutations: $EGFR^{L858R+T790M+C797S}$ (RMS); $EGFR^{19del747\_750+T790M+C797S}$ (19DMS). The values in parentheses represent the 95% confidence intervals associated with the CC_{50} values.

Supplementary Table 12.CC_{50} values of osimertinib with leelamine in A549 *EGFR*-mutant cells

Cell lines	Leelamine (μM)	CC_{50} values (nM)
19DMS	0	3120.00 (1486.00–6551.00)
19DMS	0.5	1693.00 (729.60–3928.00)
19DMS	1	29.79 (15.52–57.17)
19DMS	3	0.31 (0.10–0.98)
RMS	0	3230(1626.00–6417.00)
RMS	0.5	1029.00 (521.10–2031.00)
RMS	1	20.78 (12.39–34.83)
RMS	3	0.40 (0.16–1.00)

CC_{50}: 50% cytotoxicity concentration; EGFR: epidermal growth factor receptor. Mutations: $EGFR^{L858R+T790M+C797S}$ (RMS); $EGFR^{19del747\_750+T790M+C797S}$ (19DMS). The values in parentheses represent the 95% confidence intervals associated with the CC_{50} values.
